# *Clostridioides difficile* aggravates dextran sulfate solution (DSS)-induced colitis by shaping the gut microbiota and promoting neutrophil recruitment

**DOI:** 10.1080/19490976.2023.2192478

**Published:** 2023-03-23

**Authors:** Danfeng Dong, Tongxuan Su, Wei Chen, Daosheng Wang, YiLun Xue, Qiuya Lu, Cen Jiang, Qi Ni, Enqiang Mao, Yibing Peng

**Affiliations:** a Department of Laboratory Medicine, Ruijin Hospital, Shanghai Jiao Tong University School of Medicine, Shanghai, China; b Department of Emergency, Ruijin Hospital, Shanghai Jiao Tong University School of Medicine, Shanghai, China; c Faculty of Medical Laboratory Science, College of Health Science and Technology, Shanghai Jiao Tong University School of Medicine, Shanghai, China

**Keywords:** *Clostridioides difficile*, dextran sulfate solution (DSS)-induced colitis, gut microbiota, neutrophil, IL-1β

## Abstract

*Clostridioides difficile* is a pathogen contributing to increased morbidity and mortality of patients with inflammatory bowel disease (IBD). To determine how *C. difficile* affects the severity of colitis, we constructed a dextran sulfate solution-induced colitis model challenged with *C. difficile*. Without antibiotic administration, *C. difficile* led to transient colonization in mice with colitis, but still significantly enhanced disease severity as assessed by weight loss, histopathological damages, and inflammatory cytokine concentrations. Because this effect is independent of toxin production as shown by infection with a non-toxigenic strain, we focused on changes in the gut microbiota. The microbiota altered by *C.difficile*, featured with reduced proportions of *g_Prevotellaceae_UCG-001* and *g_Muribaculaceae*, were confirmed to contribute to disease severity in colitis mice via fecal microbiota transplantations. The inflamed colon showed neutrophil accumulation by flow cytometric analysis and myeloperoxidase immunochemical staining. There was enrichment of upregulated genes in leukocyte chemotaxis or migration as shown by RNA sequencing analysis. The isolated neutrophils from *C. difficile*-infected mice with colitis showed a robust migratory ability and had enhanced expression of cytokines and chemokines. We observed a detrimental role of neutrophils in the progress of disease by hindering neutrophil recruitment with the CXCR2 inhibitor SB225002. Furthermore, neutrophil recruitment appeared to be regulated by interleukin (IL)-1β, as inhibition of IL-1β production by MCC950 markedly ameliorated inflammation with decreased neutrophil accumulation and neutrophil-derived chemokine expression. In conclusion, our study provides information on the complicated interaction between microbiota and immune responses in *C. difficile*-induced inflammation in mice with colitis. Our findings could help determine potential therapeutic targets for patients with IBD concurrent with *C. difficile* infection.

## Introduction

*Clostridioides difficile* is a gram-positive, spore-forming, anaerobic bacterium, which is the major cause of antibiotic-associated infections in health-care facilities. Perturbation of intact microbiota or the loss of indigenous microorganisms renders individuals susceptible to *C. difficile* colonization, causing diseases, such as diarrhea and pseudomembranous colitis, or even death.^1–3^ Although antibiotic treatment is the most common risk factor for *C. difficile* infection (CDI), other factors are also recognized, especially the preexistence of inflammatory bowel disease (IBD).^4–6^ IBDs, such as Crohn’s disease (CD) and ulcerative colitis (UC), are characterized by excessive intestinal inflammation, accompanied by aberrant immune responses and dysbiosis of the gut microbiota. Patients with IBD are approximately five times more likely to develop CDI.^7^ CDI results in longer hospitalizations, increased escalation and readmission rates, a higher colectomy risk, and increased mortality of patients with IBD.^8^ These findings highlight the importance of studying IBD with concurrent CDI.

Regarding how IBD status affects subsequent infection by *C.difficile*, previous studies reported that intestinal inflammation and distinct intestinal microbiota may lead to susceptibility to CDI in the *Helicobacter hepaticus*-induced colitis mouse model.^9^ Furthermore, increased Th17 cells and Th17-related cytokines induced by colitis are responsible for the subsequent severity of CDI.^10^ However, the causes of the increased severity of IBD by *C. difficile* remain poorly understood. Generally, the progression of IBD is a complicated process with a combination of genetic and environmental factors, and host – microbe interactions.^11^ Dysbiosis of the gut microbiota, defined as decreased microbial diversity and an imbalance between potentially protective and pathogenic microorganisms, may play a major role in the pathogenesis of IBD.^12,13^ A few clinical studies have reported the effect of *C. difficile* on the microbiota of patients with IBD and showed pronounced intestinal dysbiosis in patients with concomitant IBD and CDI.^14^ However, whether this dysbiosis is truly a cause or a consequence remains unestablished.

In fragile gut environment with a disturbed microbial community, immune cells have prominent roles in maintaining intestinal hemostasis via releasing cytokines and chemokines. This in turn impairs intestinal barrier integrity and perpetuates gut inflammation.^11^ Neutrophils, as a first responder, are implicated in the development of colitis.^15^ Neutrophils communicate with gut microbes by sensing microbiota-derived components through toll-like receptors and inflammasome signaling pathways or responding to metabolites, such as short-chain fatty acids, through histone deacetylases and G protein-coupled receptors.^15–18^ After neutrophils are recruited to an inflamed colon, they defend pathogens by releasing antimicrobial peptides and reactive oxygen species, forming neutrophil extracellular traps and producing inflammatory cytokines and chemokines.^19^ Studies have shown that mice depleted of neutrophils exhibit aggravated intestinal inflammation,^20^ while excessive accumulation of neutrophils leads to the persistence of inflammatory responses and epithelial damage.^21^ These studies suggest a controversial role of neutrophils in the pathogenesis of colitis.

This study aimed to investigate how *C. difficile* affects the severity of dextran sulfate solution (DSS)-induced colitis in mice. We found that transient colonization of *C. difficile* in mice with colitis changed the gut microbiome community and increased neutrophil infiltration by upregulating multiple migration genes. The altered microbiota due to *C. difficile* was responsible for the disease severity and promoted neutrophils to express higher levels of proinflammatory cytokines and chemokines. The robust neutrophil infiltration was probably regulated by increased interleukin (IL)-1β levels because hindering the production of IL-1β by the inhibitor MCC950 significantly alleviated colitis induced by *C. difficile* in DSS mice. Our findings may help to better understand how intestinal inflammatory responses are driven by *C. difficile* in colitis and help provide evidence for potential therapeutic targets in patients with IBD and a risk of CDI.

## Results

### Exposure to C. difficile increases the disease severity of DSS-induced colitis

**Figure 1.**
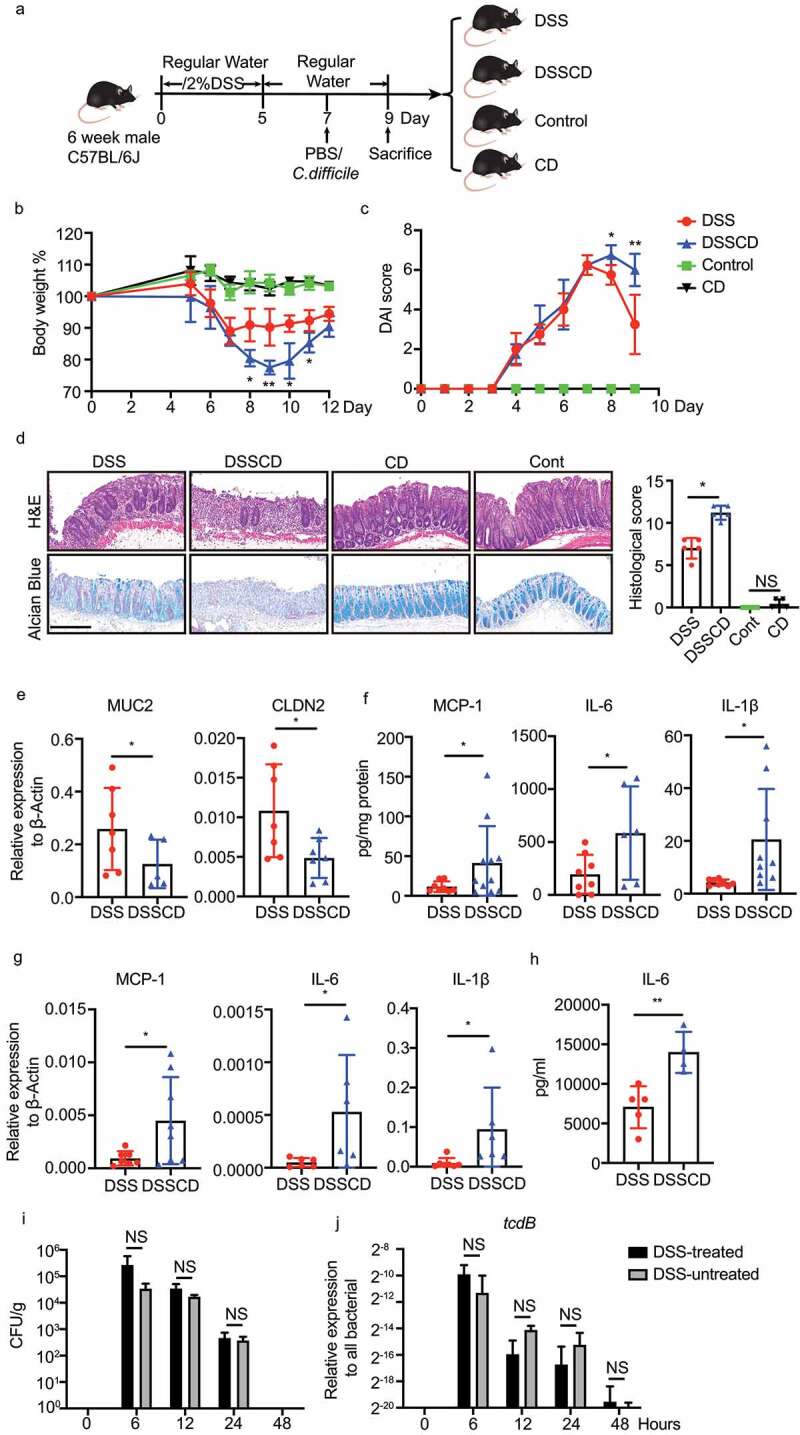
Increased disease severity in mice with DSS challenged with *C. difficile*. (a) Schematic outlining the timing and treatment regiments for each group (*n* = 5–10/group). Mice were culled and samples for further analysis were collected at 2 days post-infection. (b, c) Weight loss (b) and disease activity scores (c) in each group are shown, and significant differences between the DSS and DSSCD groups are indicated on the corresponding day (*P < 0.05, **P < 0.01). (d) Representative images of H&E and alcian blue staining of colonic tissue (left) and histological scores (right). Scale bar, 200 μm. (e – h) Relative expression levels of the gut barrier proteins MUC2 and CLDN2 (e) and the cytokines MCP1, IL-6, and IL-1β (g) were examined by real-time qPCR using actin as an internal control. Protein concentrations of MCP-1, IL-6, and IL-1β in the colon (f) and serum (h) were detected using flow cytometry. Colonic cytokine production (f) was normalized to the total protein concentration. (i and j) the *C. difficile* burden in the colonic contents of mice in different groups at different time points post-infection was measured by CFU counting through culturing (i) and real-time qPCR analysis of *tcdB* relative expression (j). Each dot indicates an individual mouse. Data are shown as the mean ± standard deviation (SD) and represent at least three independent experiments. Statistical analysis between the groups was performed by the Mann – Whitney test. *P < 0.05, **P < 0.01; NS, not significant.

First, we examined the effect of *C. difficile* on gut inflammation in mice with experimental colitis. We constructed a 7-day mouse model of DSS-induced colitis, followed by oral gavage of *C. difficile* on day 7 (Figure 1a). DSS mice challenged with the pathogen (DSSCD group) showed more severe colitis, presenting more weight loss and higher disease activity scores (Figure 1b,c). In line with the phenotype, a histological examination of colonic tissues from the DSSCD group showed increased histological scores as shown by extensive inflammation, marked epithelial disruption, and severe crypt collapse (Figure 1d). A damaged intestinal epithelial barrier in the DSSCD group was also determined by a reduced abundance of goblet cells using alcian blue staining (Figure 1d) and decreased expression levels of *muc2* and *cldn2* (Figure 1e), which are critical components of the gut barrier. Thirteen proinflammatory cytokines were measured using fluorescence-encoded beads and analyzed by a flow cytometer. The proinflammatory cytokines monocyte-chemoattractant protein (MCP)-1, IL-6, and IL-1β were remarkedly elevated at the protein and mRNA levels in the DSSCD group (Figure 1f,g, Supplementary Figure S1), along with increased serum IL-6 concentrations (Figure 1h). In contrast, a challenge with *C. difficile* in control mice (CD group) did not result in any intestinal damage or enhanced inflammatory responses (Figure 1b-d). To determine whether the pathological effect of *C. difficile* on colitis was distinctive in this mouse model, we also investigated other common pathogens, namely *Escherichia coli*, *Staphylococcus aureus*, *Enterococcus faecalis*, and *Candida albicans*, but *C. difficile* had the greatest effect as shown by weight loss (Supplementary Figure S2). Intriguingly, when we measured the *C. difficile* burden from fresh colonic contents, it was comparable between DSS-treated and untreated mice at 6, 12, 24, or 48 hours post infection (Figure 1i, j). The number of *C. difficile* cells peaked at 6 hours, and they were barely detected after 48 hours, along with similar trends detected in the transcripts of the *tcdB* gene. These data suggested that, regardless of DSS treatment, oral gavage of *C. difficile* led to a non-sustained presentation of this pathogen, which could be considered as transient colonization. This condition aggravated the disease severity and induced excessive inflammatory responses of colitis.

### Transient colonization of C. difficile alters the gut microbiota in mice with colitis

**Figure 2.**
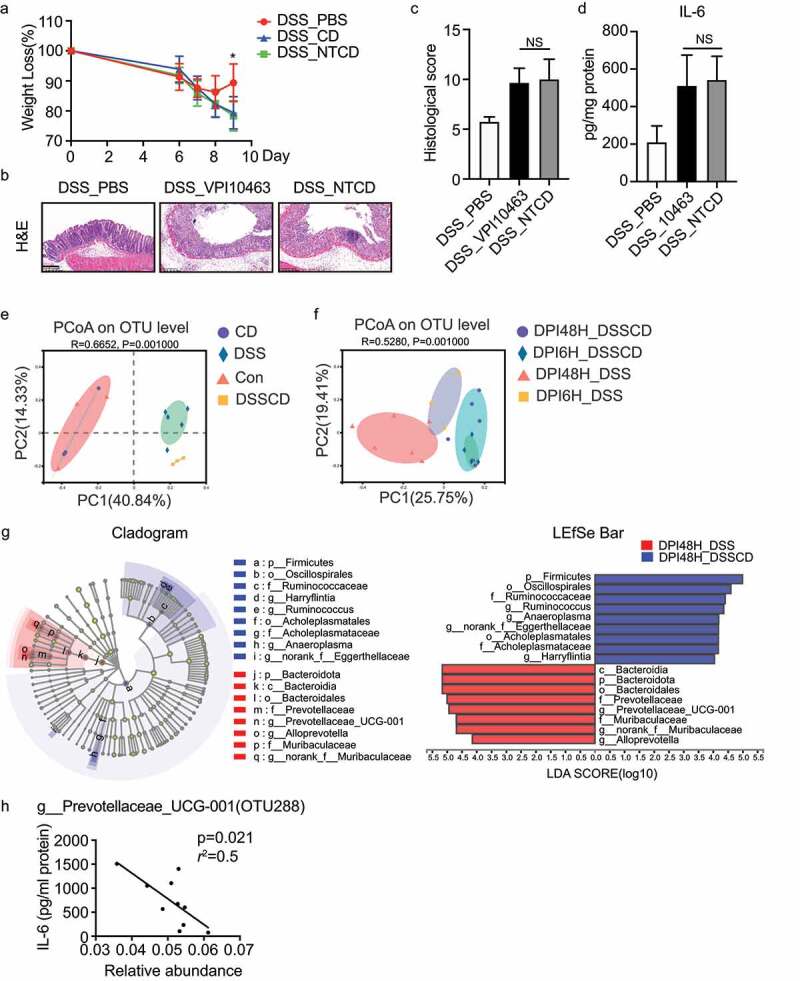
The gut microbiota is altered in mice with colitis challenged with *C. difficile*. (a – d) the effect of *C. difficile* on mice with colitis was independent of toxin production, as indicated by weight loss (a), H&E staining (b), histological scores (c), and production of IL-6 as shown by ELISA (d). Scale bar, 200 μm. (e and f) PcoA at the OTU level for samples from feces (e) and colonic contents (f), and the plots were based on the Bray – Curtis distance. The horizontal and vertical axes represent inter-sample variations. Each point represents an individual sample, and different colors refer to different groups. (g) Cladograms were generated by linear discriminant analysis effect size analysis to detect the differences in bacterial taxa between the DSSCD and DSS groups. Circles show phylogenetic levels from the phylum to the genus. To screen out differentially abundant taxa, the linear discriminant analysis threshold score was set to>4.0. Red and blue bars indicate taxa enrichment in the DPI48H_DSS and DPI48H_DSSCD groups, respectively. (h) Correlations between IL-6 levels and relative abundance of *g_prevotellaceae_ucg001* (OTU288) in DSSCD groups (DPI6H_DSSCD and DPI48_DSSCD) were analyzed by Spearman’s correlation. Each dot represents a value from an individual mouse. Data are expressed as the mean±sd. Statistical differences between groups were assessed by the Mann – Whitney test. *P < 0.05.

Toxins A and B were the main virulent factors for *C. difficile*. To determine whether pathogen-mediated inflammation was dependent on toxin production, we challenged DSS mice with VPI10463 (a hypervirulent strain) and a non-toxigenic strain (NTCD) isolated from the clinic. The NTCD strain behaved similarly to the VPI10463 strain, leading to increased disease severity and robust inflammatory responses as indicated by weight loss, colonic tissue histology and histological scores, and IL-6 concentrations (Figure 2a–d). This implied that the pathogenic effect of *C.difficile* on colitis might be independent of toxin production. Because of the importance of the gut microbiota in regulating intestinal immune responses associated with the pathophysiology of colitis, we performed 16S rRNA and internal transcribed spacer (ITS) rDNA region sequencing to characterize the bacterial and fungal community structures, respectively. After confirming the sufficient depth of sequencing coverage via rarefaction curves (Supplementary Figure S3a), we conducted beta diversity analysis of principle coordinates analysis (PCoA) of the Bray – Curtis distance to assess the variability among the groups. The fecal samples showed that bacterial communities in the DSSCD and DSS groups were clearly separated (*R* = 0.6652, *P* = 0.001), while those in the CD and control groups showed a similar microbiota structure (Figure 2e). Additionally, microbial richness and diversity were remarkably reduced in the DSS and DSSCD groups as shown by alpha diversity measured with the ACE and Shannon indices (Suplementary Figure S3a). We did not observe any significant changes in the fungal community among the four groups, as shown by beta or alpha diversity determined by PCoA (*R* = 0.116, *P* = 0.414) or the ACE or Shannon index (Suplementary Figure S3b, c). These data suggest that *C. difficile* colonization affects the gut microbiota, especially the bacterial community structure.

**Figure 3.**
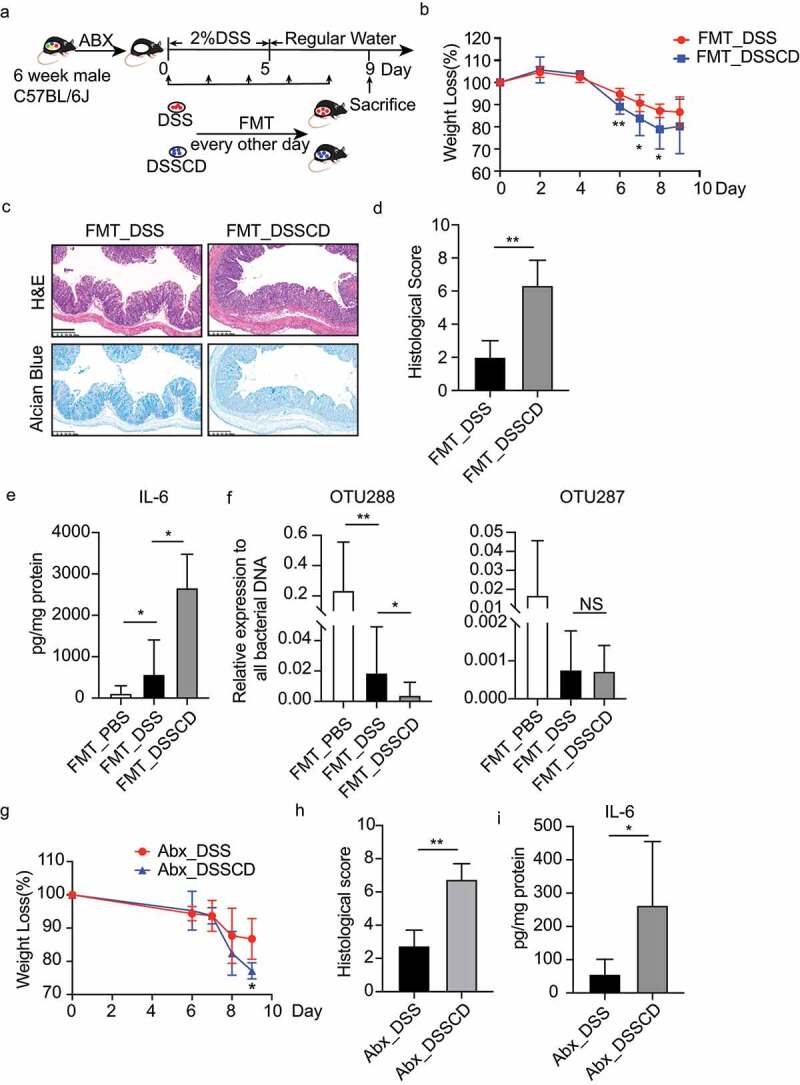
Altered gut microbiota in DSSCD group contributes to the severity of colitis. (a) Experimental design of FMT. The mice were pretreated with an antibiotic cocktail for 3 weeks, followed by 2% DSS treatment and oral gavage of fecal samples derived from DSSCD and DSS group every other day during modeling. (b – e) the severity of colitis in the mouse transplanted fecal microbiota in the DSSCD and DSS groups was assessed by body weight loss (b), H&E and alcian blue staining (c), histological scores (d), and IL-6 levels from colonic tissue (e). Scale bars, 200 μm. (f) the relative abundance of OTU288 and OTU287 was quantified by real-time qPCR with normalization to total bacterial (16S rRNA). (g – h) the effect of *C. difficile* on mice with colitis and antibiotic pretreatment was evaluated by weight loss (g), histological scores from H&E staining (h), and colonic IL-6 production by ELISA (i). Scale bars, 200 μm. Data are shown as the mean±sd and represent at least three independent experiments. Statistical analysis between groups was conducted by the Mann – Whitney test. *P < 0.05, **P < 0.01.

We then investigated the dissimilarity of bacterial communities between the DSSCD and DSS groups using colonic contents collected at 6 and 48 hours post-infection, grouped as DPI6H_DSSCD, DPI48H_DSSCD, DPI6H_DSS, and DPI48H_DSS. As shown by rarefaction curves, sequences were sufficient to analyze the bacterial diversity of the samples (Suplementary Figures 3d). PCoA analysis by the Bray-Curtis distance and weighted unifrac metrics both showed that transient colonization of *C. difficile* was a major driver of community similarity because mice challenged with *C. difficile* were clustered together and were clearly distinguishable from DSS mice (Figure 2f, Supplementary FigureS 3f). Additionally, the communities in the DPI6H_DSS and DPI48H_DSS were distinct, indicating different disease statuses of colitis. Consistently, alpha diversity including Shannon and ACE indices as well as Faith’s phylogenetic diversity and species richness also showed comparable indices in the *C. difficile* challenged group (DPI6H_DSSCD and DPI48H_DSSCD), and these indices were slightly higher than those in the DSS group (DPI6H_DSS and DPI48H_DSS) (Supplementary Figure S3e). We conducted linear discriminant analysis of the effect size (linear discriminant analysis > 4.0) to detect the predominant taxonomic differences between the groups. At 6 hours post-infection, *f_Lachnospiraceae* and *f_Oscillospirales* were more abundant in the DSSCD group than in the DSS group (Supplementary Figures 3 g). However, after 48 hours post-infection, the proportions of *p_Bacteroidota*, mainly *g_Prevotellaceae_UCG-001*, *g_Muribaculaceae*, and *g_Alloprevotella* were lower, while those of *p_Firmicutes*, including *g_Ruminococcaeceae*, *g_Anearoplasma*, and *g_Harryflintia* were relatively enriched in the DSSCD group compared with the DSS group (Figure 2g). The Wilcoxon rank sum test between the DPI48H_DSSCD and DPI48H_DSS groups at the operational taxonomic unit (OTU) level showed that OUT288 and OTU287 were the top enriched species that were different between the groups, and they corresponded to *g_Prevotellaceae_UCG001* and *f_Muribaculaceae*, respectively (Supplementary Figure S3h). To identify the prominent genus associated with inflammatory indices, we conducted a correlation analysis between MCP-1 and IL-6 concentrations and the relative abundance of genera. Among the most 20 abundant species at the OTU level, OTU288 and OTU287 showed significant negative correlations with the production of IL-6 and MCP-1 (Supplementary S3i). In multiple regression analysis models including treatment groups as variables, OTU288 was detected correlated with IL-6 levels independently (*P* = 0.041) in the DSSCD groups (DPI6H_DSSCD and DPI48H_DSSCD) (*r*^2^ = 0.5006, *P* = 0.0221) (Figure 2h), while OTU287 was found significant (*P* = 0.037) in DSS groups (DPI6H_DSS and DPI48H_DSS) (Supplementary S3j). OTU288 was also negatively correlated with the number of OTU reads of *C. difficile* (OTU418) (*r*^2^ = 0.2, *P* = 0.031) (Supplementary Figure S2k). Therefore, the gut bacterial communities that showed less abundance of *g_Prevotellaceae_UCG001* driven by *C. difficile* were probably associated with the enhanced inflammatory status of colitis.

#### C. difficile-induced changes in the microbiota contribute to the severity of colitis

To further determine the causality between *C. difficile*-related changes in the gut microbiota and disease severity, we transferred gut microbiota collected from DSSCD and DSS groups to microbiota-depleted mice through fecal microbiota transfer (FMT) assays (Figure 3a), grouped as FMT_DSSCD and FMT_DSS group, respectively. Donor drafts confirmed the absence of *C.difficile* and the differentially abundant microbes mainly *g_Prevotellaceae_UCG001* (OTU288) by 16s rRNA sequencing (data not shown). Mice in the FMT_DSSCD group showed significantly more weight loss, higher histological scores calculated by infiltration of immune cells, intestinal epithelial integrity, and crypt structure, less mucin staining, and higher IL-6 concentrations than those in the FMT_DSS group (Figure 3b–e). Consistent with the production of cytokines, the relative DNA burden of OTU288 was obviously decreased in the FMT_DSSCD group, although no significant difference was found in OTU287 between the FMT_DSSCD and FMT_DSS groups. To determine if the microbiota is a unique factor affecting disease severity, we pretreated mice with antibiotics before DSS to lessen the effect on the gut microbiome. We then performed a *C. difficile* challenge on antibiotic-treated mice and found exacerbating disease, as shown by weight loss, tissue damage, and local inflammation (Figure 3g–i). This finding indicated that the gut microbiota was not the only factor affecting disease severity. Collectively, our findings suggested that the gut microbiota affected by *C. difficile* contributed to the aggravated colitis.

#### C. difficile promotes colonic neutrophil accumulation

**Figure 4.**
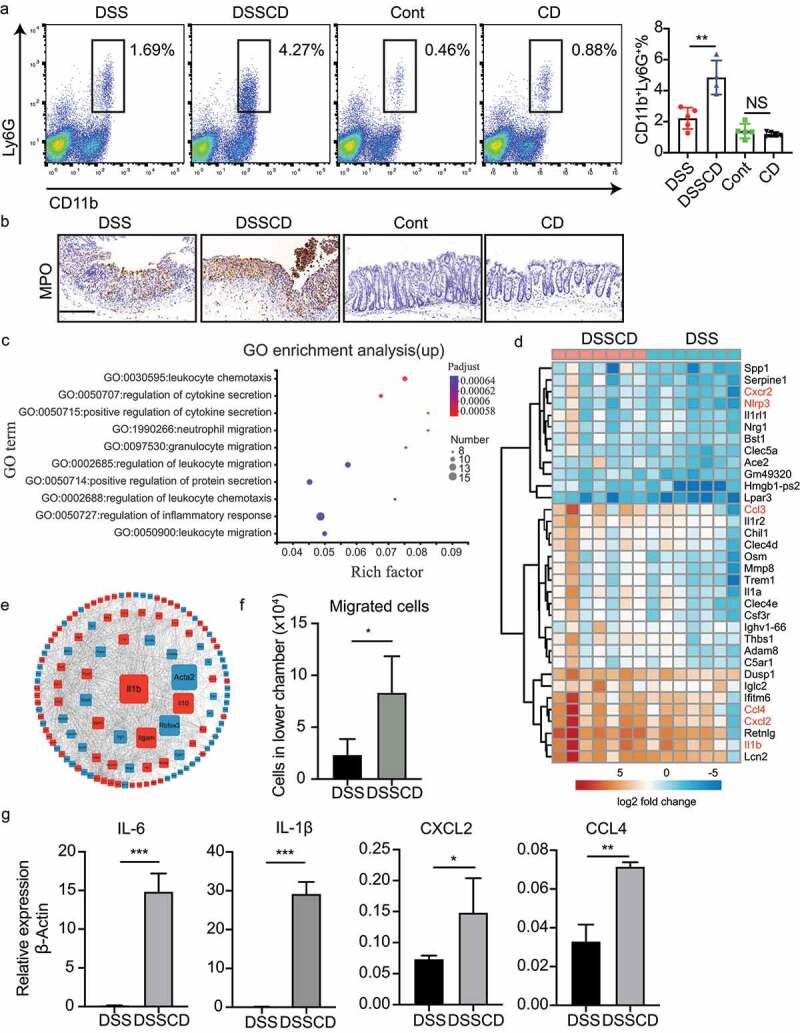
Mice with colitis challenged with *C. difficile* show increased neutrophil infiltration and enhanced expression of migratory genes. (a) Representative flow plots (left) and relative quantification (right) of the proportions of CD11b^+^Ly6G^+^ in CD45 cells. (b) Representative images of immunohistochemical MPO staining. Scale bars, 200 μm. (c) Upregulated genes in the DSSCD group relative to the DSS group were enriched for GO functional analysis. The top 10 pathways are shown in a bubble plot. The size and color of the bubble represent gene numbers enriched in each pathway and the respective enrichment significance. (d) a heatmap depicts upregulated gene expression from the most enriched pathways between the DSSCD and DSS groups. (e) a gene expression interaction network was generated by STRING and shown by Cytoscape. Red and blue rectangles represent up- and downregulated genes in the DSSCD group, respectively. The size of the rectangles indicates their betweenness centrality value. (f) Neutrophils were isolated from bone marrow in the DSSCD and DSS groups. The number of migrated neutrophils in the lower chamber was counted using trypan blue staining. (g) the mRNA transcripts of IL-6, IL-1β, CXCL2, and CCL4 in neutrophils isolated from the DSSCD and DSS groups were detected by real-time qPCR. Gene expression was normalized to β-actin. Data are shown as the mean±sd. Each dot indicates an individual mouse. Data (a, b, f, and g) are representative of at least three independent experiments. Statistical analysis between the groups was performed by the Mann – Whitney test. *P < 0.05, **P < 0.01, ***P < 0.001.

The intestinal microbiota is considered a critical regulator of intestinal immunity. To better understand immune responses to *C. difficile*-related alteration of the microbiota in DSS mice, we characterized the accumulation of relevant immune cells, such as macrophages, neutrophils, and CD4^+^ T cell subtypes, from the lamina propria. The proportions of macrophages (CD11b^+^F4/80^+^) and CD4^+^ T cells, along with the main CD4^+^T subsets Th17 (CD4^+^IL17^+^) and T_reg_ (CD4^+^Foxp3^+^), were comparable between DSSCD and DSS groups (Supplementary Figure S4a-d). Noticeably, we detected a robust increase in the proportion of neutrophils (CD11b^+^Ly6G^+^) driven by *C. difficile* in mice with colitis (Figure 4a), which was further suggested by myeloperoxidase (MPO) staining of colonic tissue (Figure 4b). Therefore, we focused on neutrophils to investigate how they are regulated by persistent colitis induced by *C. difficile*.

To determine how the host responds to *C. difficile*-induced inflammation leading to the accumulation of neutrophils, we performed RNA sequencing and compared the gene expression of colonic tissues between the DSSCD and DSS groups. Overall, there were 290 upregulated and 206 downregulated genes that were differentially expressed in the DSSCD group relative to the DSS group (Supplementary Figure S5a). Samples from each group were distinctly clustered by these differentially expressed genes, which suggested that host responses in colitis were changed by *C. difficile* (Supplementary Figure S5b). We then performed enrichment analysis of differentially expressed gene sets using Gene Ontology (GO) biological terms. The pathways of downregulated genes were mostly enriched in immune responses related to monocytes, such as monocyte chemotaxis, lymphocyte migration, and mononuclear cell migration, and interferon beta responses (Supplementary Figure S5c). Strikingly, the enrichment of upregulated genes was mainly involved in neutrophil migration and chemotaxis, as well as cytokine secretion (Figure 4c), which was particularly consistent with phenotypes of neutrophil infiltration. Furthermore, we identified upregulated genes from the most enriched pathways and plotted them in a heatmap (Figure 4d). The transcription levels of genes responsible for neutrophil migration, such as the inflammasome pathway genes *nlrp3* and *Il1-β*, and chemokines such as *cxcl2*, *cxcr2*, *ccl3*, and *ccl4*, were significantly increased (Figure 4d). Moreover, the protein interaction network of differentially expressed genes by STRING analysis showed that IL-1β might be the core protein interacting with differential expression genes (DEGs) (Figure 4e). Based on the above-mentioned findings of upregulated genes related to neutrophil recruitment, we aimed to determine the migratory capacity of neutrophils isolated from DSSCD and DSS groups using trans-well assays. More cells from the DSSCD group than those from the DSS group were transmitted from the upper chamber, which was attached to the reverse side of the membrane (Supplementary Figure S5d) and moved to the bottom (Figure 4f). These cells displayed robust trafficking ability. Moreover, neutrophils isolated from the DSSCD group also showed remarkably higher *Il-6*, *Il1-β, Cxcl2*, and *Ccl4* mRNA expression than those from the DSS group, indicating a highly proinflammatory effect. Taken together, these findings suggest that *C. difficile*-aggravated colitis is characterized by accumulated neutrophils, and it shows a strong ability of migration and proinflammatory mediator production.

### An altered microbiota by C. difficile leads to the production of proinflammatory cytokines and chemokines in neutrophils and macrophages

**Figure 5.**
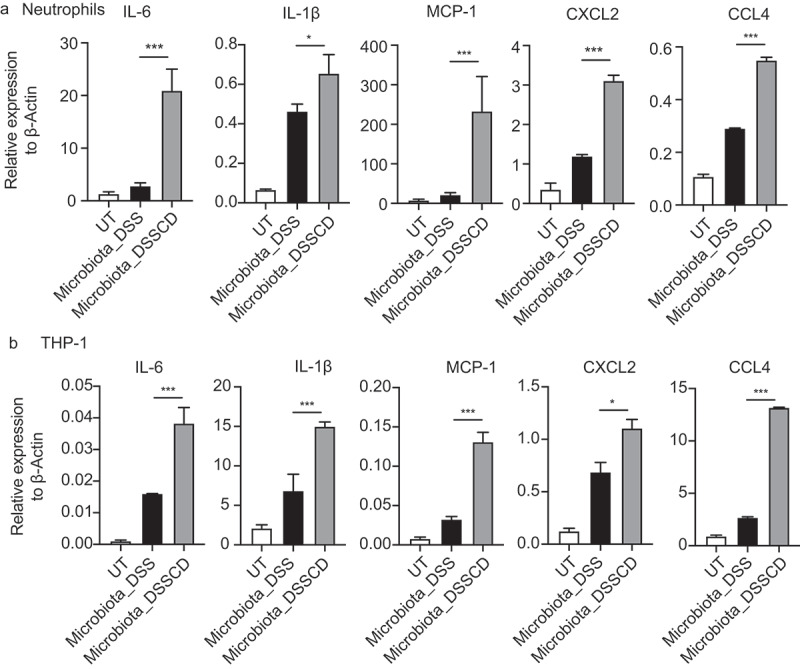
Gut microbiota in DSSCD mice induces neutrophils and THP-1 cells, leading to higher levels of proinflammatory cytokines and chemokines. Neutrophils (a) and THP-1 cells pretreated with PMA (b) were stimulated *in vitro* in the presence or absence of microbiota suspensions prepared from different groups for 3 hours. The mRNA levels of IL-6, IL-1β, MCP-1 CXCL2, and CCL4 were measured by real-time qPCR and normalized to β-actin. Statistical analysis was performed by the Mann – Whitney test. *P < 0.05, **P < 0.01, ***P < 0.001. Results are representative of at least three independent experiments.

Neutrophils, which are a critical component of innate immunity, produce inflammatory cytokines and chemokines in an inflamed intestine by sensing microbial components and metabolites. Therefore, cross-talk between neutrophils and microbiota is considered essential in the gut microenvironment. We investigated whether the altered microbiota by *C. difficile* contributes to increased concentrations of inflammatory mediators *in vitro*. Fecal contents from DSSCD and DSS groups were collected and subjected to stimulation of neutrophils isolated from mice with colitis. We found that the proinflammatory cytokines IL-6, MCP-1, and IL-1β, and the chemokines Chemokine (C-X-C motif) ligand 2(CXCL2) and C-C Motif Chemokine Ligand 4 (CCL4) were significantly more highly expressed in neutrophils upon stimulation of the microbiota in DSSCD mice than in DSS mice (Figure 5a). Neutrophils can also be activated by these cytokines and chemokines released by macrophages. Therefore, we also assessed the effect of the microbiota on THP-1 cells, which is a human monocytic cell line differentiated into macrophages, in the presence of phorbol-12-myristate-13-acetate (PMA). As expected, the microbiota in the DSSCD group showed remarkably stimulated THP-1 cells, which expressed higher IL-6, IL-1β and MCP-1, CXCL2, and CCL4 concentrations. Taken together, these data demonstrated that the gut microbiota affected by *C. difficile* promoted the expression of genes related to inflammation and neutrophil migration.

### Inhibition of neutrophil migration attenuates colitis in DSS mice challenged with C. difficile

**Figure 6.**
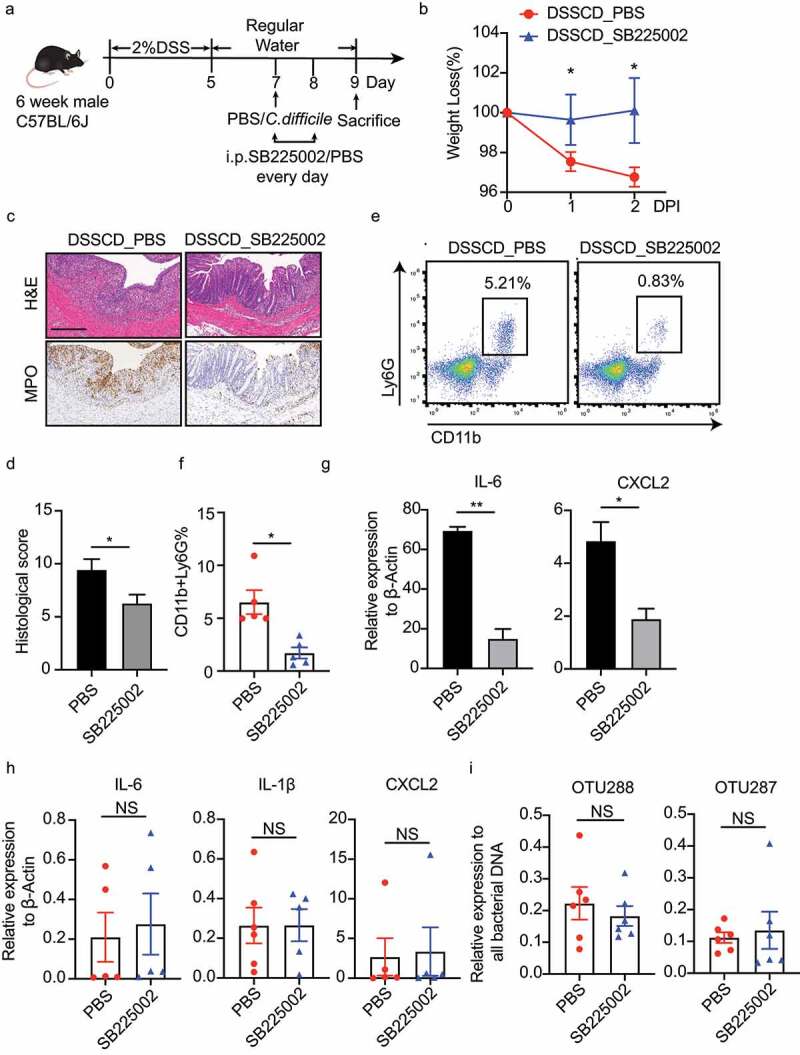
Inhibition of neutrophil migration by SB225002 alleviates colitis driven by *C. difficile*. (a) Schematic diagram of the CXCR2 inhibition model. After DSS mice were challenged with or without *C. difficile* on day 7, the mice were injected i.P. with the CXCR2 inhibitor SB225002 at a dose of 1 mg/kg/mouse on days 7 and 8, and PBS was used as a negative control. (b – d) the severity of colitis was assessed by weight loss (b), H&E and MPO staining of colonic sections (c), and histological scores (d). Scale bars, 200 μm. (e and f) Representative flow cytometric plots (e) and a histogram (f) quantifying the proportions of CD11b^+^Ly6G^+^ in CD45^+^ cells. (g and h) Expression of IL-6, IL-1β, and CXCL2 mRNA was detected by real-time qPCR in isolated neutrophils (g) and colonic tissue (h) with or without inhibitor treatment. (i) Colonic contents were collected to detect the relative abundance of OTU288 and OTU287 as shown by real-time qPCR. DNA expression was normalized to total bacteria (16S rRNA). Significant differences were determined by the Mann – Whitney test. *P < 0.05, **P < 0.01, ***P < 0.001. Data are shown as the mean±sd and are representative of at least three independent experiments. i.P., intraperitoneally; DPI, days post-infection; NS, no significance.

Because DSS mice challenged with *C. difficile* showed increased infiltration of neutrophils triggered by an altered gut microbiota, we then aimed to determine the role of leukocyte migration in the process of colitis. CXCR2 is a chemokine receptor expressed in neutrophils, and it plays a crucial role in recruiting neutrophils and activating related chemokines. We then constructed a colitis model with *C.difficile* using the CXCR2 inhibitor SB225002 to hinder the migratory capacity of neutrophil (Figure 6a). The mice treated with SB225002 developed milder colitis post-infection of *C. difficile*, with less weight loss, less tissue damage as shown by hematoxylin and eosin (H&E) staining, and lower histopathological scores (Figure 6b, d). Consistently, we observed decreased numbers of MPO-positive cells by immunostaining (Figure 6c) and a reduced proportion of neutrophils (CD11b^+^Ly6G^+^) by flow cytometry, accompanied by decreased neutrophil-derived IL-6 and CXCL2 expression (Figure 6e, f). Interestingly, blockage of CXCR2 did not affect the colonic transcription levels of *Il-6*, *Il-1β, Cxcl2*, *Mcp-1*, or *Ccl4* (Figure 6g, Supplementary Figure S6b), or the intestinal abundance of OTU288 and OTU287 (Figure 6h). However, the CXCR2 inhibitor SB225002 did not affect the diseases of colitis alone (Figure 6a, Supplementary FigureS 6a). Our data suggested that the inhibition of neutrophil migration effectively alleviated *C. difficile*-mediated disease in mice with colitis, without changing levels of inflammation cytokines and the proportions of predominant species.

### IL-1β secretion may lead to neutrophil accumulation during colitis challenged with C. difficile

**Figure 7.**
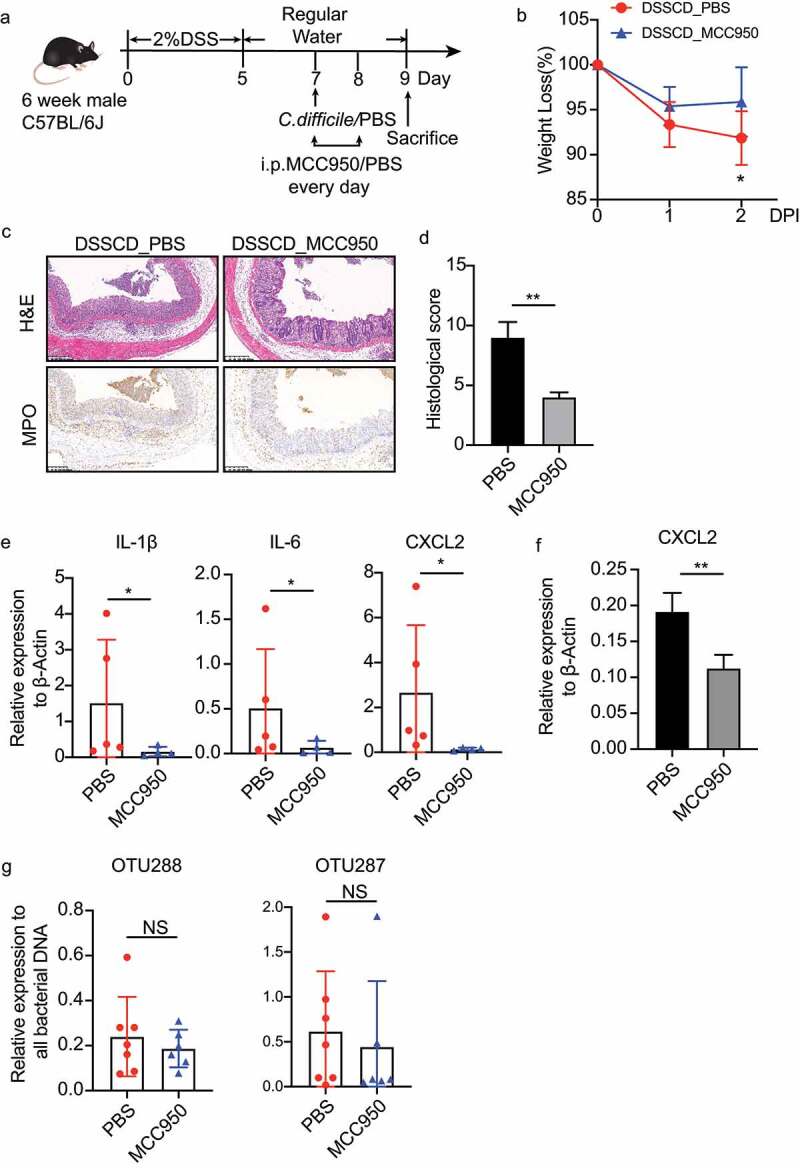
Inhibition of IL-1β by MCC950 ameliorates the severity of colitis challenged with *C. difficile*. (a) Schematic diagram of the MCC950 inhibition model. After DSS mice were challenged with *C. difficile* on day 7, they were injected i.P. with MCC950 at a dose of 20 mg/kg/mouse on days 7 and 8, and PBS was used as a negative control. (b – d) Disease severity was assessed by weight loss (b), H&E staining (c), and histological sores (d). (e and f) Expression of IL-1β, IL-6, and CXCL2 mRNA in colonic tissue (e) and CXCL2 mRNA in isolated neutrophils (f) were detected by real-time qPCR and normalized to β-actin. (g) the relative DNA burden of OTU288 and OTU297 was measured by real-time qPCR and normalized to total bacteria (16s rRNA). Significant differences were determined by the Mann – Whitney test. *P < 0.05, **P < 0.01, ***P < 0.001. Data are shown as the mean±sd and are representative of at least three independent experiments. i.P., intraperitoneally; DPI, days post-infection; NS, no significance.

Since IL-1β was involved in leukocyte chemotaxis in GO biological terms, played a main role in interacting with DEGs as shown by protein interaction analysis (Figure 4e), and its production was not affected by neutrophil accumulation (Figure 6g), we then hypothesized to examine whether IL-1β is implicated in regulating neutrophil recruitment and intestinal inflammation during colitis. We constructed a colitis mouse model with or without a *C. difficile* challenge using MCC950, which is a selective NLRP3 inflammasome inhibitor, to inhibit the production of IL-1β *in vivo* (Figure 7a). MCC950 treatment significantly diminished the pathogenic effect of *C. difficile* on colitis, as evaluated by milder weight loss and histological pathology of colonic tissues (Figure 7b–d), but it did not affect the severity of colitis alone (Supplementary Figure S7). As expected, reduced neutrophil infiltration in the colon with less MPO-positive cells was observed (Figure 7c). In addition, colonic transcription of IL-1β, IL-6, and CXCL2 was consistently lower (Figure 7e), along with a decreased production of CXCL2 in neutrophils with MCC950 treatment (Figure 7f). We also detected the relative expression of OTU288 and OTU287, but no changes were found with MCC950 treatment. Taken together, our findings suggested that elevated IL-1β expression driven by *C. difficile* in colitis contributed to neutrophil infiltration and intestinal inflammatory responses.

## Discussion

*C. difficile* has a pathogenic effect on patients with IBD, with severe symptoms and poor clinical outcomes. The association between IBD and CDI regarding pathogenic susceptibility, changes in the microbiota, and host immune responses is so complicated that applying an appropriate murine model is necessary to clarify this association. Researchers have constructed a colitis model concurrent with CDI by antibiotic administration to disturb the indigenous microbiota and make it susceptible for colonization.^10,22^ Antibiotics are not a necessary prescription for patients with IBD and whether they are an independent risk factor for concurrent CDI remains controversial.^23,24^ Therefore, the pretreatment of antibiotics in mouse models is unlikely to represent the clinical situation. Therefore, we challenged mice with DSS-induced colitis with *C. difficile* in the absence of antibiotics. Notably, we found that although the *C. difficile* challenge to non-antibiotic colitis failed to lead to persistent colonization, it still considerably aggravated the disease status. The minor difference between our study and Zhou et al.’s report, which showed no worsened histopathological damage, might be attributed to the different concentrations of DSS used for colitis.^22^ Moreover, studies have shown that intestinal inflammation along with an altered microbiota in colitis are beneficial for *C. difficile* colonization in the IL10^−/−^ colitis mouse model.^9^ However, in our study, we found that treatment with DSS did not significantly affect the colonization status of *C. difficile*, but mice with only colitis challenged with *C. difficile* developed much more severe intestinal inflammation. This finding suggested that the underlying intestinal microenvironment of colitis, other than *C. difficile* itself, was a prerequisite for disease progression. Additionally, it warns that exposure to a *C. difficile*-contaminated environment puts patients with IBD at risk of severe symptoms, even if they are non-persistent colonizers. By comparing to other commonly detected gut pathogens, *C.difficile* had a greater pathogenic effect on colitis, emphasizing the importance to make further investigations on the mechanisms by which *C.difficile* aggravated colitis.

Accumulating studies have indicated that the gut microbiota participates in the pathogenesis of IBD.^25^ Patients with IBD and CDI harbor more-pronounced intestinal dysbiosis, but the observed features do not clearly indicate whether CDI expedites the intestinal dysbiosis or patients with disturbed gut microbiota are more prone to CDI.^14,26^ In this study using a dynamic analysis of bacterial communities post-infection, we found that a surge of *C. difficile* at 6 hours increased the microbial diversity with a higher abundance of *Lachnospirales* and *Oscillospiraceae* (previously known as *Ruminococcaceae*). *Lachnospiraceae* and *Oscillospiraceae* families are considered potentially protective, producing short-chain fatty acids that provide energy sources to colonic enterocytes and secondary bile acids to hinder the growth of *C. difficile*.^26–29^ Similar results were also described from clinical studies, which showed that the emergence of *C. difficile* might cause a beneficial transient change in bacterial taxa.^30,31^ Subsequently, at the height of the disease (48 hours post-infection) when *C. difficile* cells had already been cleared, bacterial communities in DSSCD mice were characterized by a lower abundance of *g_Prevotellaceae_UCG-001* (OTU288) and *g_Muribaculaceae* (OTU287), which belong to the Bacteroidetes phylum. The declined abundance of *g_Prevotellaceae*_UCG001 was independently related to the disease severity and showed a highly inflammatory effect on neutrophils and macrophages in colitis mice with *C.difficile*. Members of *Bacteroidetes* degrade polysaccharides,^32^ supplying nutrients to other residents in the gut and producing metabolites to exert an anti-inflammatory effect.^33^ Therefore, we propose that they have a protective effect on colitis. However, we could not determine the exact role of either genus. Collectively, the influx of *C. difficile* might induce protective microbiota responses, but the microbiota still accelerates gut inflammation in colitis.

Disturbed gut microbial communities, accompanied by uncontrolled host immune responses, are major drivers of disease complications.^11^ An inflamed colon driven by *C. difficile* in the colitis mouse model shows accumulation of neutrophils, in addition to enriched gene sets associated with the migration ability. Consequently, researchers have paid attention to the function of neutrophils. In patients with IBD, the degree of neutrophil infiltration and the reappearance of neutrophils in the intestinal mucosa are thought to represent the disease activity and clinical remission, respectively.^34,35^ During the whole process of DSS modeling, inhibiting neutrophil infiltration daily by blocking CXCR2, a common ligand for neutrophil functionality, was reported to decrease proinflammatory cytokine production and reduced intestinal damages^36^. However, since neutrophil infiltration and inflammatory cytokine production in DSS models were initiated one-day post treatment^37^, blockade of CXCR2 when colitis was developed presently showed limited effect on inflammation status induced by DSS, but significantly alleviated disease severity induced by *C.difficile*. This finding indicated a detrimental role of colonic neutrophils in the present colitis mouse model challenged with *C. difficile*, despite their reported protective role in attacking invasion of pathogens.^38^ MPO staining of colonic tissues reflected reduced levels of reactive oxygen species, which are considered hazardous to tissues by oxidative DNA damage to epithelial cells.^39^ Neutrophil-derived cytokines and chemokines were also decreased, which are closely related to the initiation and continuation of inflammation by regulating innate and adaptive immune responses.^40^ CXCR2 blockade probably inhibited its cross-talk with other immune cells. Moreover, the unchanged levels of colonic inflammatory mediators, such as IL-6, IL-1β, and CXCL2, indicated neutrophils’ downstream act in the intestinal inflammations.

Using RNA sequencing analysis, we mapped the differentially expressed genes enriched in leukocyte chemotaxis or migration, especially IL-1β and CXCL2, CXCR2, CCL3, and CCL4. Furthermore, IL-1β was a core gene, which showed the most intricate interactions with other genes, and may be an upstream regulator of neutrophil recruitment. Though IL-1β is important for repair of intestinal epithelial cell and reconstitution of the epithelial barrier^41^, the excessive production of IL-1β may exacerbate colon inflammation and is associated with intestinal inflammation of IBD and the pathogenesis of CDI.^42–44^ However, the role of IL-1β in IBD with CDI is undetermined. Our study showed a central role of IL-1β in regulating neutrophil functions and the colonic inflammatory status in *C.difficile-*driven inflammations in colitis. MCC950 was considered to reduce IL-1β release by blocking NLRP3-dependent ASC oligomerization and NLRP3 inflammasome assembly^45^, meanwhile it could also decrease the mRNA expressions of IL-1β^46^. Since IL-1β expression could be regulated by IL-1β itself and multiple cytokines^47^, the reduced transcriptional levels by MCC950 could be attributed to the inhibited secretion of IL-1β or IL-1β downstream cytokines. Inhibiting IL-1β production by MCC950 failed to relieve the symptoms of colitis when it was used after colitis induced but significantly ameliorated the colitis with subsequent *C.difficile* invasion. Hopefully, IL-1β antagonists may be therapeutic agents for patients with IBD and a risk of CDI. How IL-1β is affected by *C. difficile* in colitis needs to be clarified. *C. difficile* in cells and toxins can trigger the activation of inflammasomes,^44,48,49^ releasing IL-1β and mediating intestinal inflammation. The altered microbiota affected by *C. difficile* in our study also promoted IL-1β production *in vitro*. Since the colonization of *C. difficile* in our model is transient, whether IL-1β was induced by *C. difficile* directly, indirectly by the microbial community, or both synergistically is unknown. IL-1β might regulate the neutrophils by driving Th17 differentiation to induce emergency granulopoiesis or by upregulating the neutrophil chemoattractant to enhance chemotaxis.^50,51^ In this study, IL-1β appeared to regulate the expression of several chemokines, such as CXCL2, which is a ligand for CXCR2 involved in triggering neutrophil function and recruitment. Recently, Pavlidis et al. predicted IL-1β as an important driver of neutrophil-active chemokines via IL-22 pathways through bioinformatics analysis.^52^. Nevertheless, the precise mechanism regarding how IL-1β is involved in *C. difficile*-induced inflammation in colitis requires further investigation.

In summary, our study shows the pathogenic effect of transient colonization of *C. difficile* in mice with DSS-induced colitis. We conducted a comprehensive investigation on the interaction between the gut microbiota and host immune responses. We highlight the importance of changes in the microbiota, IL-1β production, and neutrophil accumulation, and propose a possible pathway of microbiota – IL-1β–neutrophil regulation in *C. difficile*-driven gut inflammation in DSS-induced colitis. Our findings will hopefully be helpful in determining potential therapeutic targets to treat patients with IBD concurrent with CDI.

## Materials and methods

### Bacterial strains and culture conditions

*C. difficile* VPI10463 (ATCC 43,255), *Escherichia coli* (ATCC 25,922), *Staphylococcus aureus* (ATCC 25,923), *Enterococcus faecalis* (ATCC 29,212), *Candida albicans* (ATCC 90,028), and THP-1 cells (ATCC TIB-202) were purchased from the American Type Culture Collection (ATCC). The non-toxigenic strain NTCD was isolated clinically and identified as negative for toxin genes, such as *tcdA*, *tcdB*, *cdtA*, and *cdtB*, previously in our laboratory.^53^ THP-1 cells were cultured in RPMI 1640 medium supplemented with 10% fetal bovine serum (Gibco, USA) and 50 mg/L penicillin/streptomycin (Gibco, USA). *C. difficile* strains were cultured in brain heart infusion (Oxoid Ltd., USA) broth supplemented with 5 g/L yeast extract (Oxoid Ltd.), 0.1% L-cysteine (Sangon Biotech, China), and 0.1% sodium taurocholate (Sangon Biotech, China) for 48 hours at 37°C anaerobically. The other bacterial strains and *C. albicans* were cultured in Luria-Bertani (Sangon Biotech, China) and yeast peptone dextrose broth (Sangon Biotech, China), respectively, for 24 hours aerobically. The liquid culture was centrifuged at 1500 g for 5 minutes, and the pellet was washed twice with sterile phosphate-buffered saline (PBS). The inoculum was adjusted to approximately 5 × 10^6^ Colony Forming Units (CFU)/ml.

### DSS-induced colitis and pathogen challenge

Male mice aged 6 weeks were purchased from Charles River (Beijing, China) and housed at a constant temperature of 20°C–22°C with a 12-hour light–dark circle under specific pathogen-free conditions. The mice were acclimatized for 1 week before modeling. The experimental schema is shown in Figure 1a. To induce acute colitis, the mice were administered 2% (w/v) DSS (molecular weight: 36000–50,000 Da, MP Biomedicals) in drinking water for 5 days, followed by the administration of regular water. To construct IBD concurrent with *C. difficile* or other pathogen models, the mice were administered with 10^6^ CFUs by oral gavage in random order on day 7. Four groups were used for further monitoring, namely the DSS (DSS treated mice without *C.difficile* challenge), DSSCD (DSS treated mice challenged with *C. difficile*), control (DSS untreated mice without *C. difficile* administration), and CD (DSS untreated mice challenged with *C. difficile* only) groups. To perform CXCR2 and NLRP3 inhibition in the *in vivo* model, the DSSCD group were administered the CXCR2 selective inhibitor SB225002 (Sigma Aldrich, USA) (1 mg/kg/mouse) or the NLRP3 selective inhibitor MCC950 (Selleck, USA) (20 mg/kg/mouse) intraperitoneally (i.p.) on days 7 and 8 (Figures 6a and 7a). During the modeling, the mice were weighed and scored daily. Disease activity scores were assessed by weight loss, stool consistency, and bleeding as described previously^54^.Samples including stools, serum, and colonic tissue and contents were collected on day 9, at 2 days post*-C. difficile* treatment, unless specified otherwise. To monitor the burden of *C. difficile in vivo*, the mice were scarified at 6, 12, 24, and 48 hours post-infection, and colonic contents were freshly collected for further analysis. All animal experiments were approved by the Ethics Committee of Ruijin Hospital, Shanghai Jiaotong University School of Medicine.

### Quantification of the C. difficile burden

To quantify the *C. difficile* burden, colonic contents were suspended in ethanol, serially diluted, and plated anaerobically at 37°C on brain heart infusion broth supplemented with 5 g/L yeast extract, 0.1% L-cysteine, 0.1% sodium taurocholate, 16 mg/L cefoxitin (BBI Life Science, USA) and 500 mg/L D-cycloserine (BBI Life Science, USA). After 48 hours of incubation, CFUs were counted and normalized to the stool weight.

### Histological analysis

Tissue samples were harvested from the colon, rinsed gently with PBS to remove colonic content, and fixed in 4% paraformaldehyde. Embedded samples were stained with H&E and alcian blue, and MPO immunostaining was also performed^55^. Scores ranging from 0 to 3 were used to assess epithelial disruption, crypt architecture, and the degree and range of inflammatory cell infiltration^56^. This assessment was made by at least two independent, blinded observers. Images were captured by a Nikon DS-F2 microscope.

### Tissue protein and cytokine analysis

Colonic tissues were isolated and homogenized in 1 ml of PBS with a protease inhibitor cocktail (Roche, USA). The homogenate was centrifuged at 12,000 g for 5 minutes, and the supernatants were collected and stored at−80°C. The protein concentrations were measured using the Enhanced BCA Protein Assay Kit (Beyotime, China). The production of proinflammatory cytokines, namely IL-23, IL-1⍺, interferon-γ, tumor necrosis factor-⍺, MCP-1, IL-12, IL-1β, IL-10, IL-6, IL-27, IL-17A, interferon-β, and Granulocyte-macrophage colony-stimulating factor (GM-CSF), was detected by flow cytometry using the FACSCantoII (BD, USA) with fluorescence-encoded beads in accordance with the manufacturer’s instructions of the LEGENDplex Mouse Inflammation Panel (Biolegend, USA). The production of IL-6 was also measured by the enzyme-linked immunosorbent assay (ELISA) kit (Biolegend, USA). Cytokine concentrations were normalized to the total protein concentration.

### DNA extraction and real-time quantitative polymerase chain reaction

DNA was extracted from fresh colonic contents using the TIANamp Stool DNA Kit (Tiangen Biotech, China) in accordance with the manufacturer’s instructions. The expression levels of the *tcdB* gene and the relative abundance of OTU288 (*g_Prevotellaceae_UCG-001*) and OTU287 (*g_Muribaculaceae*) were quantified by performing real-time quantitative polymerase chain reaction (qPCR) using TB Green™ Premix Ex Taq™ (Takara, Japan) on a Light Cycler 480 Real-Time PCR system (Roche). The data were normalized to total bacteria (16S rRNA). The primer sequences are listed in Supplementary Table S1.

### RNA extraction and real-time qPCR

Total RNA from tissue or cell samples was extracted with TRIzol Reagent (Invitrogen, USA). Complementary DNA was synthesized by the PrimeScript RT Reagent Kit (Takara). Real-time qPCR was conducted as described above, using β-actin as the internal control. The primers are listed in Supplementary Table S1.

### Lamina propria cell isolation and flow cytometry

To isolate mononuclear cells in the lamina propria, tissues were incubated with PBS supplemented with 1 mM DL-dithiothreitol (Sigma Aldrich, USA) for 30 minutes in an incubator at 37°C. This was followed by another 30 minutes’ treatment in PBS supplemented with 30 mM Ethylenediaminetetraacetic acid (EDTA) (BBI Life Science, China). The tissues were then cut into small pieces and digested in the RPMI 1640 medium (Gibco, USA) with 10% fetal bovine serum (Gibco, USA), 200 U/ML collagenase (Sigma Aldrich), and 150 μg/ML deoxyribonuclease (Sigma Aldrich, USA) for 1 hour. After the cells were strained through 40-μm filters, they were obtained in the 40% to 80% interface of Percoll (Sigma Aldrich, USA) by density gradient centrifugation. To perform flow cytometric analysis, 10^6^ cells were resuspended in fluorescence-activated cell sorting buffer comprising PBS with 0.5% bovine serum albumin (BBI Life Science, China) and 2 mM EDTA. After blocking the Fc receptor with anti-mouse CD16/32 and staining with the viability dye Zombie (Biolegend, USA), the following antibodies were stained for surface markers: FITC-CD45.2, PerCP/Cyanine5.5-CD11b, PerCP/Cyanine5.5-CD4, APC-CD25, PE-Ly-6 G, and APC-F4/80. To perform Treg analysis, cells were fixed and permeabilized with Fix/Perm solution (Biolegend, USA) for 30 minutes after staining with surface markers, and then incubated with PE-Foxp3 antibody. To detect IL-17A, cells were stimulated with cell activation cocktail (Biolegend, USA) in RPMI 1640 medium for 5 hours, followed by surface marker labeling. The cells were then treated with a transcription factor staining buffer set (BD Biosciences, USA) in accordance with the manufacturer’s protocols and were stained with PE-IL-17A. All antibodies used in this study were purchased from Biolegend, USA. Flow cytometric data were acquired on a BD Canto II flow cytometer (BD Biosciences) and were analyzed using Flowjo software 10.0.

### 16s rRNA and ITS1 sequencing

Genomic DNA was extracted from colonic contents as described above. The DNA quality and concentration were determined with a NanoDrop ND-2000 spectrophotometer (Thermo Scientific, USA). DNA was sent to Majorbio Bio-Pharm Technology Co. Ltd. (Shanghai, China) for amplification, sequencing, and data processing as described previously.^31^ Briefly, the hypervariable V3–V4 regions were amplified with the primer pairs 338F (5′-ACTCCTACGGGAGGCAGCAG-3′) and 806 R (5′-GGACTACHVGGGTWTCTAAT-3′) for 16s rRNA sequencing. The fungal ITS1 was amplified with the primer pairs ITS1F (5′-CTTGGTCATTTAGAGGAAGTAA-3′) and ITS2R (5′-GCTGCGTTCTTCAT CGATGC-3′) for fungal sequencing. Amplicons were paired-end sequenced on an Illumina MiSeq PE300 platform (San Diego, CA) in accordance with the standard protocols. Raw FASTQ files were de-multiplexed using an in-house per script, quality-filtered by fastp version 0.19.6, and then merged by FLASH version 1.2.7. The optimized sequences were clustered into OTUs with 97% sequence similarity and removed chimera using UPARSE 7.1^57^. The taxonomy of each OTU representative sequence was analyzed by RDP Classifier version 2.2 against a 16S rRNA gene database (e.g., Silva v138) or the Targeted Host Fungi ITS1 database using a confidence threshold of 0.7. All bioinformatic analyses were performed by the Majorbio Cloud Platform (https://cloud.majorbio.com/).

### RNA sequencing and transcriptomic analysis

RNA derived from colonic tissue was extracted as described above. An RNA sequencing transcriptome library was prepared by following the TruSeqTM RNA sample preparation kit from Illumina. Briefly, mRNA was isolated in accordance with the polyA selection method by oligo(dT) beads and then fragmented by fragmentation buffer. Double-stranded cDNA was synthesized using the SuperScript double-stranded cDNA synthesis kit (Invitrogen) with random hexamer primers. Libraries were then size selected for cDNA target fragments of 300 base pairs on 2% Low Range Ultra Agarose followed by PCR amplification using Phusion DNA polymerase (NEB, USA) for 15 PCR cycles. After quantification by TBS380, paired-end RNA sequencing was performed with the Illumina HiSeq xten/NovaSeq 6000 sequencer. The raw paired-end reads were trimmed, and the poor quality reads were removed by SeqPrep (https://github.com/jstjohn/SeqPrep) and Sickle (https://github.com/najoshi/sickle), respectively, with default parameters. Clean reads were separately aligned to reference the genome with the orientation mode using HISAT2 (http://ccb.jhu.edu/software/hisat2/index.shtml) software and assembled by StringTie (https://ccb.jhu.edu/software/stringtie/index.shtml?t=example). Gene abundance was quantified by RSEM (http://deweylab.biostat.wisc.edu/rsem/), and a differential expression analysis was performed using differentially expressed genes with |log_2_Fold Change (FC)|>1. In addition, a functional enrichment analysis of GO was performed to identify which differentially expressed genes were significantly enriched in GO terms at a Bonferroni-corrected *p* value ≤ 0.05 compared with the whole transcriptomic background. This analysis was carried out by Goatools (https://github.com/tanghaibao/Goatools). All bioinformatic analyses were performed using the Majorbio Cloud Platform.

### Antibiotic treatment and FMT

To clear the gut microbiota, mice received antibiotic cocktails containing 1 g/L ampicillin, 1 g/L metronidazole, 0.5 g/L vancomycin, and 1 g/L neomycin for 2 weeks in drinking water. This was followed by another week of administration of 2 g/L streptomycin, 0.17 g/L ciprofloxacin, 0.125 g/L gentamicin, and 1 g/L bacteriocin. After antibiotic treatment, stool samples of mice were collected and cultured on Columbia blood agar plate (Chromagar, China) anaerobically and aerobically to confirm microbiota depletion. Subsequently, the mice were modeled with 2% DSS as described above. Fecal transplant samples were collected at day 9, 2 days post infection, prepared using colonic contents pooled from DSSCD and DSS group donor mice (*n* = 5–10/group) and stored at −80°C. 16s rRNA sequencing was analyzed for transplant donor samples to assess the microbial composition and confirm the absence of *C.difficile*. On the day of transplantation, samples were resuspended in sterile PBS at a concentration of 100 mg/mL, and the supernatants were collected after centrifugation at 800 rpm for 3 minutes. Transplantation should be completed with fresh supernatants by oral gavage within 10 mins to minimize changes in microbial compositions^58^. Antibiotic-treated mice were administered 200 μL of PBS suspensions in each mouse by oral gavage every other day from the start of DSS modeling.

### Neutrophil isolation, transmigration assay, and cellular stimulation

Neutrophils were collected from mouse bone marrow as described previously.^59^ Briefly, bone marrow cells were harvested in the RPMI 1640 medium supplemented with 10% fetal bovine serum and 1% penicillin/streptomycin. The neutrophils were purified from the interface between Hisotopague 1119 (Sigma Aldrich, USA) and Histopaque 1077 (Sigma Aldrich, USA) by density gradient centrifugation. The cells were counted with trypan blue and identified by flow cytometry staining with anti-CD45, anti-CD11b, and anti-Ly6G. In the transmigration assay, approximately 2 × 10^5^ cells in 200 μl of medium were seeded above the transmigration membrane, while culture medium supplemented with 20% fetal bovine serum was added to the basolaterial side. After incubation for 24 hours, migrated cells in the lower chamber were collected for counting. Migrated cells on the membrane of the basolateral side were fixed in 4% paraformaldehyde for 30 minutes, followed by staining with 0.1% crystal violet (Sangon Biotech). Images were acquired by optical microscopy. To perform stimulation assays, microbiota samples from the DSS and DSSCD groups were prepared in the same manner as that for FMT donors. THP-1 cells were seeded on 12-well plates and incubated with 100 ng/ML PMA (Sigma Aldrich) for 1 day, and then cultured by fresh medium without PMA for another 24 hours. Approximately, 10^6^ isolated neutrophil cells or THP-1 cells in each well were incubated with 50 μl of microbiota PBS suspension for 3 hours. The cells were collected and subjected for further analysis.

### Statistical analysis

Statistical analyses were conducted using SPSS software version 16.0, and *P* < 0.05 was considered significant. Data were generated using Graphpad Prism software version 8.0 and R software version 4.0.5. Statistical differences between the two groups were assessed using Welch’s t-test or the Mann–Whitney test depending on whether the data showed normal distribution Rarefaction curves were generated to assess the sufficiency of sequence reads to describe the bacterial diversity and rarified to lowest OTUs per sample. Alpha diversity including the Shannon and ACE indices as well as Faith’s phylogenetic diversity and species richness were calculated at the OTU level and compared among the groups using the Student’s t-test or paired t-test. The difference in PCoA of the Bray – Curtis distance and weighted unifrac metrics was compared by Adonis analysis. The predominant phyla or genera of the linear discriminant analysis effect size were compared using the Wilcoxon rank-sum test or Wilcoxon signed-rank test. Correlations between species relative abundance and cytokine levels were calculated using Spearman’s analysis. Since the elevated cytokines could also be affected by treatment groups, we performed multiple-regression analysis with results from four groups (DPI6H_DSSCD, DPI48H_DSSCD, DPI6H_DSS, and DPI48H_DSS), DSSCD groups (DPI6H_DSSCD and DSS48H_DSSCD), and DSS groups (DPI6H_DSS and DPI48H_DSS) separately, involving abundances of OTU288 and OTU287, treatment groups, and levels of IL-6 as variables using SPSS version 24.0.

## Supplementary Material

Supplemental Material

## Data Availability

The sequence dataset generated from this study has been deposited in the NCBI database under BioProject number PRJNA897872 in https://www.ncbi.nlm.nih.gov/bioproject/. The data that support the findings of this study are available from the corresponding author upon reasonable request.

## References

[1] Schnizlein MK, Young VB. Capturing the environment of the Clostridioides difficile infection cycle. Nat Rev Gastroenterol Hepatol. 2022;19(8):508–23. doi:10.1038/s41575-022-00610-0. In eng. 35468953

[2] Baumgartner M, Pfrunder-Cardozo KR, Hall AR. Microbial community composition interacts with local abiotic conditions to drive colonization resistance in human gut microbiome samples. Proc Biol Sci. 2021;288(1947):20203106. doi:10.1098/rspb.2020.3106. In eng. 33757361 PMC8059542

[3] Rodriguez C, Van Broeck J, Taminiau B, Delmée M, Daube G. Clostridium difficile infection: early history, diagnosis and molecular strain typing methods. Microb Pathog. 2016;97:59–78. doi:10.1016/j.micpath.2016.05.018. In eng. 27238460

[4] Kucharzik T, Ellul P, Greuter T, Rahier JF, Verstockt B, Abreu C, Albuquerque A, Allocca M, Esteve M, Farraye FA, et al. ECCO guidelines on the prevention, diagnosis, and management of infections in inflammatory bowel disease. J Crohns Colitis. 2021;15(6):879–913. doi:10.1093/ecco-jcc/jjab052. 33730753

[5] Ananthakrishnan AN. Clostridium difficile infection: epidemiology, risk factors and management. Nat Rev Gastroenterol Hepatol. 2011;8(1):17–26. doi:10.1038/nrgastro.2010.190. In eng. 21119612

[6] Rao K, Higgins PD. Epidemiology, diagnosis, and management of clostridium difficile infection in patients with inflammatory bowel disease. Inflamm Bowel Dis. 2016;22(7):1744–1754. doi:10.1097/mib.0000000000000793. In eng. 27120571 PMC4911291

[7] Singh H, Nugent Z, Yu BN, Lix LM, Targownik LE, Bernstein CN. Higher incidence of clostridium difficile infection among individuals with inflammatory bowel disease. Gastroenterology. 2017;153(2):430–438.e2. doi:10.1053/j.gastro.2017.04.044. In eng. 28479377

[8] Balram B, Battat R, Al-Khoury A, D’aoust J, Afif W, Bitton A, Lakatos PL, Bessissow T. Risk factors associated with clostridium difficile infection in inflammatory bowel disease: a systematic review and meta-analysis. J Crohns Colitis. 2019;13(1):27–38. doi:10.1093/ecco-jcc/jjy143. 30247650

[9] Abernathy-Close L, Barron MR, George JM, Dieterle MG, Vendrov KC, Bergin IL, Young VB. Intestinal inflammation and altered gut microbiota associated with Inflammatory bowel disease render mice susceptible to clostridioides difficile colonization and infection. mBio. 2021;12(3):e0273320. doi:10.1128/mBio.02733-20. 34126769 PMC8262858

[10] Saleh MM, Frisbee AL, Leslie JL, Buonomo EL, Cowardin CA, Ma JZ, Simpson ME, Scully KW, Abhyankar MM, Petri WA. Colitis-induced Th17 cells increase the risk for severe subsequent clostridium difficile infection. Cell Host Microbe. 2019;25(5):756–765 e5. doi:10.1016/j.chom.2019.03.003. 31003940 PMC6509008

[11] Neurath MF. Targeting immune cell circuits and trafficking in inflammatory bowel disease. Nat Immunol. 2019;20(8):970–979. doi:10.1038/s41590-019-0415-0. 31235952

[12] Lavelle A, Sokol H. Gut microbiota-derived metabolites as key actors in inflammatory bowel disease. Nat Rev Gastroenterol Hepatol. 2020;17(4):223–237. doi:10.1038/s41575-019-0258-z. 32076145

[13] Ott SJ, Musfeldt M, Wenderoth DF, Hampe J, Brant O, Fölsch UR, Timmis KN, Schreiber S . Reduction in diversity of the colonic mucosa associated bacterial microflora in patients with active inflammatory bowel disease. Gut. 2004;53(5):685–693. In eng. doi:10.1136/gut.2003.025403. 15082587 PMC1774050

[14] Sokol H, Jegou S, McQuitty C, Straub M, Leducq V, Landman C, Kirchgesner J, Le Gall G, Bourrier A, Nion-Larmurier I. Specificities of the intestinal microbiota in patients with inflammatory bowel disease and Clostridium difficile infection. Gut Microbes. 2018;9(1):55–60. doi:10.1080/19490976.2017.1361092. 28786749 PMC5914915

[15] Uhlig HH, Powrie F. Translating immunology into therapeutic concepts for inflammatory bowel disease. Annu Rev Immunol. 2018;36:755–781. doi:10.1146/annurev-immunol-042617-053055. In eng. 29677472

[16] Hayashi F, Means TK, Luster AD. Toll-like receptors stimulate human neutrophil function. Blood. 2003 In eng;102(7):2660–2669. doi:10.1182/blood-2003-04-1078. 12829592

[17] Furusawa Y, Obata Y, Fukuda S, Endo TA, Nakato G, Takahashi D, Nakanishi Y, Uetake C, Kato K, Kato T. Commensal microbe-derived butyrate induces the differentiation of colonic regulatory T cells. Nature. 2013;504(7480):446–450. In eng. doi:10.1038/nature12721. 24226770

[18] Koh A, De Vadder F, Kovatcheva-Datchary P, Bäckhed F. From dietary fiber to host physiology: short-chain fatty acids as key bacterial metabolites. Cell. 2016 In eng;165(6):1332–1345. doi:10.1016/j.cell.2016.05.041. 27259147

[19] Zhang D, Frenette PS. Cross talk between neutrophils and the microbiota. Blood. 2019 In eng;133(20):2168–2177. doi:10.1182/blood-2018-11-844555. 30898860 PMC6524562

[20] Kühl AA, Kakirman H, Janotta M, Dreher S, Cremer P, Pawlowski NN, Loddenkemper C, Heimesaat MM, Grollich K, Zeitz, M. Aggravation of different types of experimental colitis by depletion or adhesion blockade of neutrophils. Gastroenterology. 2007;133(6):1882–1892. In eng. doi:10.1053/j.gastro.2007.08.073. 18054560

[21] Fournier BM, Parkos CA. The role of neutrophils during intestinal inflammation. Mucosal Immunol. 2012;5(4):354–366. doi:10.1038/mi.2012.24. 22491176

[22] Zhou F, Hamza T, Fleur AS, Zhang Y, Yu H, Chen K, Heath JE, Chen Y, Huang H, Feng H. Mice with inflammatory bowel disease are susceptible to clostridium difficile infection with severe disease outcomes. Inflamm Bowel Dis. 2018;24(3):573–582. doi:10.1093/ibd/izx059. 29462386 PMC5936643

[23] Voth E, Solanky D, Loftus EV Jr., Pardi DS, Khanna S. Novel risk factors and outcomes in inflammatory bowel disease patients with Clostridioides difficile infection. Therap Adv Gastroenterol. 2021;14:1756284821997792. doi:10.1177/1756284821997792. In eng. PMC7958162 33786065

[24] Yanai H, Nguyen GC, Yun L, Lebwohl O, Navaneethan U, Stone CD, Ghazi L, Moayyedi P, Brooks J, Bernstein CN, et al. Practice of gastroenterologists in treating flaring inflammatory bowel disease patients with clostridium difficile: antibiotics alone or combined antibiotics/immunomodulators? Inflamm Bowel Dis. 2011;17(7):1540–1546. doi:10.1002/ibd.21514. 21674710

[25] Xavier RJ, Podolsky DK. Unravelling the pathogenesis of inflammatory bowel disease. Nature. 2007 In eng;448(7152):427–434. doi:10.1038/nature06005. 17653185

[26] Vacca M, Celano G, Calabrese FM, Portincasa P, Gobbetti M, De Angelis M. The controversial role of human gut lachnospiraceae. Microorganisms. 2020;8(4). doi:10.3390/microorganisms8040573. PMC7232163 32326636

[27] Gu X, Sim JXY, Lee WL, Cui L, Chan YFZ, Chang ED, Teh YE, Zhang AN, Armas F, Chandra F. Gut Ruminococcaceae levels at baseline correlate with risk of antibiotic-associated diarrhea. iScience. 2022;25(1):103644. doi:10.1016/j.isci.2021.103644. 35005566 PMC8718891

[28] Lee YJ, Arguello ES, Jenq RR, Littmann E, Kim GJ, Miller LC, Ling L, Figueroa C, Robilotti E, Perales M-A, et al. Protective factors in the intestinal microbiome against clostridium difficile infection in recipients of allogeneic hematopoietic stem cell transplantation. J Infect Dis. 2017;215(7):1117–1123. In eng. doi:10.1093/infdis/jix011. 28498996 PMC5426375

[29] Morrison DJ, Preston T. Formation of short chain fatty acids by the gut microbiota and their impact on human metabolism. Gut Microbes. 2016 In eng;7(3):189–200. doi:10.1080/19490976.2015.1134082. 26963409 PMC4939913

[30] Vincent C, Miller MA, Edens TJ, Mehrotra S, Dewar K, Manges AR. Bloom and bust: intestinal microbiota dynamics in response to hospital exposures and Clostridium difficile colonization or infection. Microbiome. 2016;4:12. doi:10.1186/s40168-016-0156-3. In eng. 26975510 PMC4791782

[31] Wang D, Dong D, Wang C, Cui Y, Jiang C, Ni Q, Su T, Wang G, Mao E, Peng Y. Risk factors and intestinal microbiota: clostridioides difficile infection in patients receiving enteral nutrition at intensive care units. Crit Care. 2020;24(1):426. doi:10.1186/s13054-020-03119-7. 32660525 PMC7359293

[32] Thomas F, Hehemann JH, Rebuffet E, Czjzek M, Michel G. Environmental and gut bacteroidetes: the food connection. Front Microbiol. 2011;2:93. doi:10.3389/fmicb.2011.00093. In eng. 21747801 PMC3129010

[33] Zafar H, Saier MH Jr. Gut bacteroides species in health and disease. Gut Microbes. 2021;13(1):1–20. doi:10.1080/19490976.2020.1848158. PMC7872030 33535896

[34] Brazil JC, Louis NA, Parkos CA. The role of polymorphonuclear leukocyte trafficking in the perpetuation of inflammation during inflammatory bowel disease. Inflamm Bowel Dis. 2013;19(7):1556–1565. doi:10.1097/MIB.0b013e318281f54e. In eng. 23598816 PMC4110963

[35] Magro F, Doherty G, Peyrin-Biroulet L, Svrcek M, Borralho P, Walsh A, Carneiro F, Rosini F, de Hertogh G, Biedermann L, et al. ECCO position paper: harmonization of the approach to ulcerative colitis histopathology. J Crohn's Colitis. 2020;14(11):1503–1511. In eng. doi:10.1093/ecco-jcc/jjaa110. 32504534

[36] Zhu F, He H, Fan L, Ma C, Xu Z, Xue Y, Wang Y, Zhang C, Zhou G. Blockade of CXCR2 suppresses proinflammatory activities of neutrophils in ulcerative colitis. Am J Transl Res. 2020;12(9):5237–5251. In eng. 33042416 PMC7540107

[37] Yan Y, Kolachala V, Dalmasso G, Nguyen H, Laroui H, Sitaraman SV, Merlin D. Temporal and spatial analysis of clinical and molecular parameters in dextran sodium sulfate induced colitis. PLoS One. 2009;4(6):e6073. In eng. doi:10.1371/journal.pone.0006073. 19562033 PMC2698136

[38] Zhou G, Yu L, Fang L, Yang W, Yu T, Miao Y, Chen M, Wu K, Chen F, Cong Y, et al. CD177 + neutrophils as functionally activated neutrophils negatively regulate IBD. Gut. 2018;67(6):1052–1063. In eng. doi:10.1136/gutjnl-2016-313535. 28468761

[39] Pereira C, Grácio D, Teixeira JP, Magro F. Oxidative stress and DNA damage: implications in inflammatory bowel disease. Inflamm Bowel Dis. 2015;21(10):2403–2417. doi:10.1097/mib.0000000000000506. In eng. 26193347

[40] Mantovani A, Cassatella MA, Costantini C, Jaillon S. Neutrophils in the activation and regulation of innate and adaptive immunity. Nat Rev Immunol. 2011;11(8):519–531. doi:10.1038/nri3024. In eng. 21785456

[41] Bersudsky M, Luski L, Fishman D, White RM, Ziv-Sokolovskaya N, Dotan S, Rider P, Kaplanov I, Aychek T, Dinarello CA. Non-redundant properties of IL-1α and IL-1β during acute colon inflammation in mice. Gut. 2014;63(4):598–609. In eng. doi:10.1136/gutjnl-2012-303329. 23793223

[42] Shouval DS, Biswas A, Kang YH, Griffith AE, Konnikova L, Mascanfroni ID, Redhu NS, Frei SM, Field M, Doty AL. Interleukin 1β mediates intestinal inflammation in mice and patients with interleukin 10 receptor deficiency. Gastroenterology. 2016;151(6):1100–1104. In eng. doi:10.1053/j.gastro.2016.08.055. 27693323 PMC5124405

[43] Coccia M, Harrison OJ, Schiering C, Asquith MJ, Becher B, Powrie F, Maloy KJ. IL-1β mediates chronic intestinal inflammation by promoting the accumulation of IL-17A secreting innate lymphoid cells and CD4+ Th17 cells. J Exp Med. 2012 In eng;209(9):1595–1609. doi:10.1084/jem.20111453. 22891275 PMC3428945

[44] Ng J, Hirota SA, Gross O, Li Y, Ulke–Lemee A, Potentier MS, Schenck LP, Vilaysane A, Seamone ME, Feng H, et al. Clostridium difficile toxin–Induced inflammation and intestinal injury are mediated by the inflammasome. Gastroenterology. 2010;139(2):542–552. 552.e13In eng. doi:. 552.e13In eng. doi:10.1053/j.gastro.2010.04.005. 20398664

[45] Coll RC, Hill JR, Day CJ, Zamoshnikova A, Boucher D, Massey NL, Chitty JL, Fraser JA, Jennings MP, Robertson AAB, et al. MCC950 directly targets the NLRP3 ATP-hydrolysis motif for inflammasome inhibition. Nat Chem Biol. 2019;15(6):556–559. In eng. doi:10.1038/s41589-019-0277-7. 31086327

[46] Perera AP, Fernando R, Shinde T, Gundamaraju R, Southam B, Sohal SS, Robertson AAB, Schroder K, Kunde D, Eri R. MCC950, a specific small molecule inhibitor of NLRP3 inflammasome attenuates colonic inflammation in spontaneous colitis mice. Sci Rep. 2018 In eng;8(1):8618. doi:10.1038/s41598-018-26775-w. 29872077 PMC5988655

[47] Fenton MJ. Review: transcriptional and post-transcriptional regulation of interleukin 1 gene expression. Int J Immunopharmacol. 1992;14(3):401–411. doi:10.1016/0192-0561(92)90170-p. In eng. 1618594

[48] Liu YH, Chang YC, Chen LK, Su P-A, Ko W-C, Tsai Y-S, Chen Y-H, Lai H-C, Wu C-Y, Hung Y-P, et al. The ATP-P2X(7) signaling axis is an essential sentinel for intracellular clostridium difficile pathogen-induced inflammasome activation. Front Cell Infect Microbiol. 2018;8:84. In eng. doi:10.3389/fcimb.2018.00084. 29616195 PMC5864904

[49] Xu H, Yang J, Gao W, Li L, Li P, Zhang L, Gong YN, Peng X, Xi JJ, Chen S. Innate immune sensing of bacterial modifications of Rho GTPases by the Pyrin inflammasome. Nature. 2014;513(7517):237–241. In eng. doi:10.1038/nature13449. 24919149

[50] Yoshimura T, Matsushima K, Oppenheim JJ, Leonard EJ. Neutrophil chemotactic factor produced by lipopolysaccharide (LPS)-stimulated human blood mononuclear leukocytes: partial characterization and separation from interleukin 1 (IL 1). J Immunol. 1987 In eng;139(3):788–793. doi:10.4049/jimmunol.139.3.788. 3298433

[51] Pyrillou K, Burzynski LC, Clarke MCH. Alternative pathways of IL-1 activation, and its role in health and disease. Front Immunol. 2020;11:613170. doi:10.3389/fimmu.2020.613170. 33391283 PMC7775495

[52] Pavlidis P, Tsakmaki A, Pantazi E, Li K, Cozzetto D, Digby- Bell J, Yang F, Lo JW, Alberts E, Sa ACC, et al. Interleukin-22 regulates neutrophil recruitment in ulcerative colitis and is associated with resistance to ustekinumab therapy. Nat Commun. 2022;13(1):5820. doi:10.1038/s41467-022-33331-8. 36192482 PMC9530232

[53] Dong D, Zhang L, Chen X, Jiang C, Yu B, Wang X, Peng Y. Antimicrobial susceptibility and resistance mechanisms of clinical Clostridium difficile from a Chinese tertiary hospital. Int J Antimicrob Agents. 2013;41(1):80–84. doi:10.1016/j.ijantimicag.2012.08.011. 23148985

[54] Taghipour N, Molaei M, Mosaffa N, Rostami-Nejad M, Asadzadeh Aghdaei H, Anissian A, Azimzadeh P, Zali MR. An experimental model of colitis induced by dextran sulfate sodium from acute progresses to chronicity in C57BL/6: correlation between conditions of mice and the environment. Gastroenterol Hepatol Bed Bench. 2016;9(1):45–52. In eng. 26744614 PMC4702041

[55] Fischer AH, Jacobson KA, Rose J, Zeller R. Hematoxylin and eosin staining of tissue and cell sections. CSH Protoc. 2008;2008:db.prot4986. doi:10.1101/pdb.prot4986. In eng. 21356829

[56] Dieleman LA, Palmen MJ, Akol H, Bloemena E, Peña AS, Meuwissen SGM, Van Rees EP. Chronic experimental colitis induced by dextran sulphate sodium (DSS) is characterized by Th1 and Th2 cytokines. Clin Exp Immunol. 1998 In eng;114(3):385–391. doi:10.1046/j.1365-2249.1998.00728.x. 9844047 PMC1905133

[57] Rc E. UPARSE: highly accurate OTU sequences from microbial amplicon reads. Nat Methods. 2013;10(10):996–998. doi:10.1038/nmeth.2604. In eng. 23955772

[58] Wu J, Wei Z, Cheng P, Qian C, Xu F, Yang Y, Wang A, Chen W, Sun Z, Lu Y. Rhein modulates host purine metabolism in intestine through gut microbiota and ameliorates experimental colitis. Theranostics. 2020;10(23):10665–10679. In eng. doi:10.7150/thno.43528. 32929373 PMC7482825

[59] Swamydas M, Luo Y, Dorf ME, Lionakis MS. Isolation of mouse neutrophils. Curr Protoc Immunol. 2015;110(3):20 1–3 20 15. doi:10.1002/0471142735.im0320s110. PMC4574512 26237011

