# Supplementary material for: Clostridioides difficile aggravates dextran sulfate solution (DSS)-induced colitis by shaping the gut microbiota and promoting neutrophil recruitment
Source: Gut Microbes. 2023 Mar 23;15(1):2192478. doi: 10.1080/19490976.2023.2192478 (PMC10038061; doi:10.1080/19490976.2023.2192478)
Supplement: Supplemental Material [file KGMI_A_2192478_SM0498.zip › Revised Supplementary Files.docx]

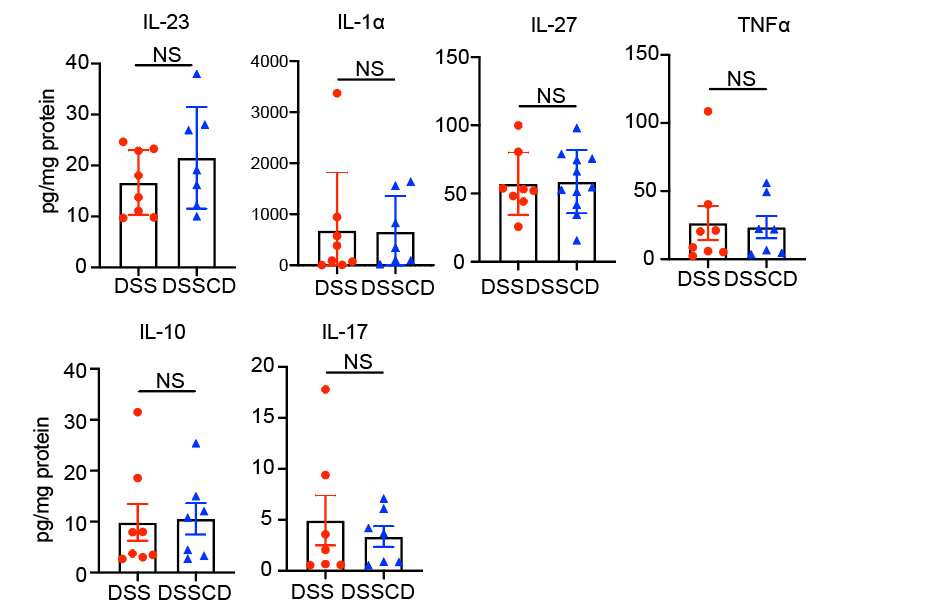


Supplementary Figure S1 Levels of IL-23, IL-1⍺, IL-27, TNF⍺, IL-10 and IL-17 from colonic tissues were measured with LEGENDplex Mouse Inflammation Panel by flow cytometry and were normalized to total protein concentration. Data are shown as mean ± standard deviation (SD) and represent at least three independent experiments. Each dot indicated an individual sample from each group. Statistical analysis between groups was performed by Mann-Whitney test. NS, not significant.


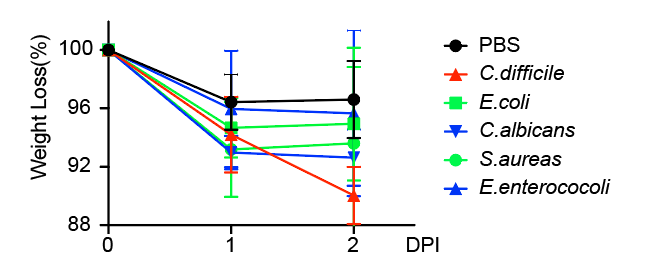


Supplementary Figure S2 The weight loss of colitis mice challenged with PBS, *C.difficile*, *E. coli*, *C.albicans*, *S.aureas*, and *E.enterococoli* was shown. DPI, days post infection. Data are shown as mean ± SD and represent at least three independent experiments.


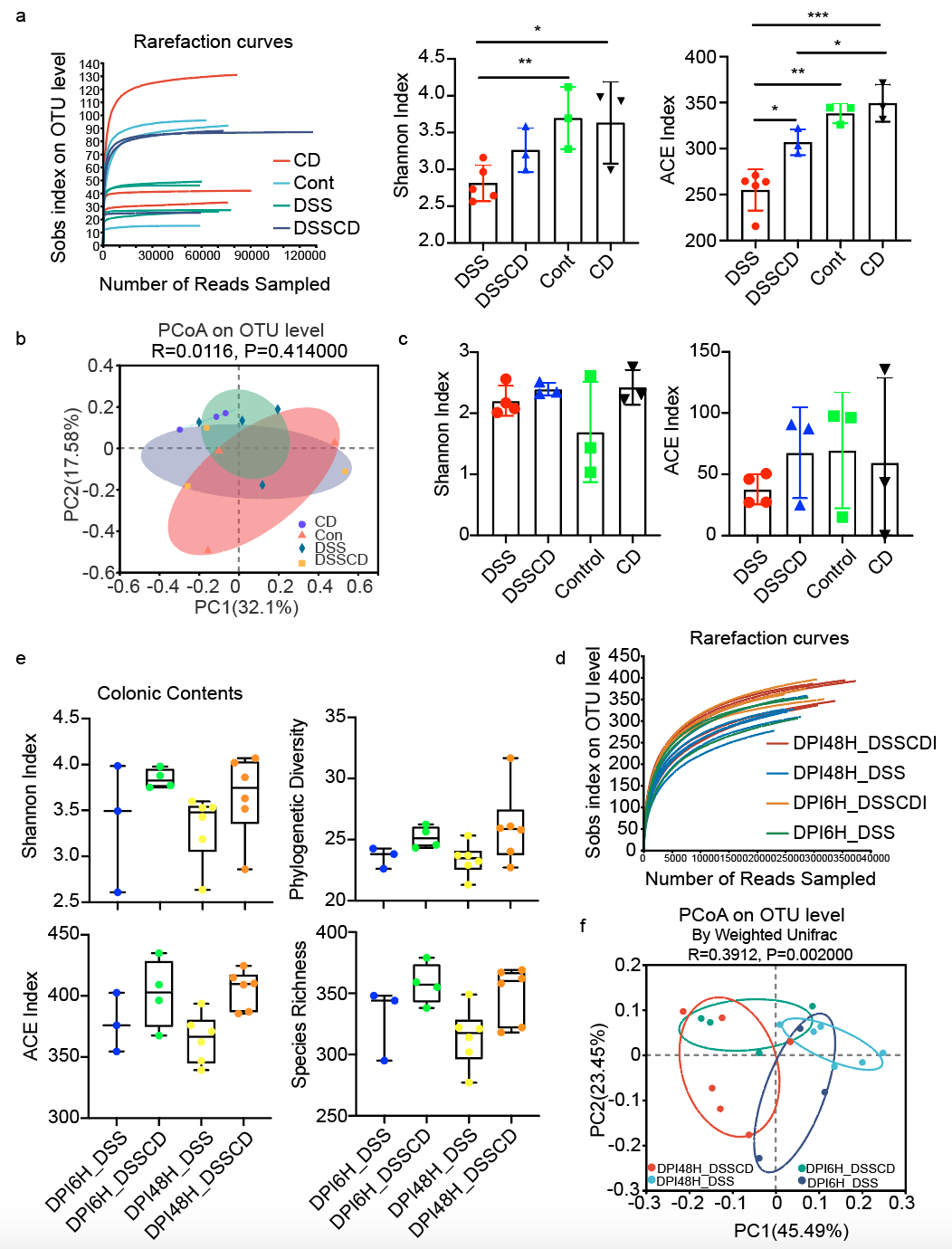


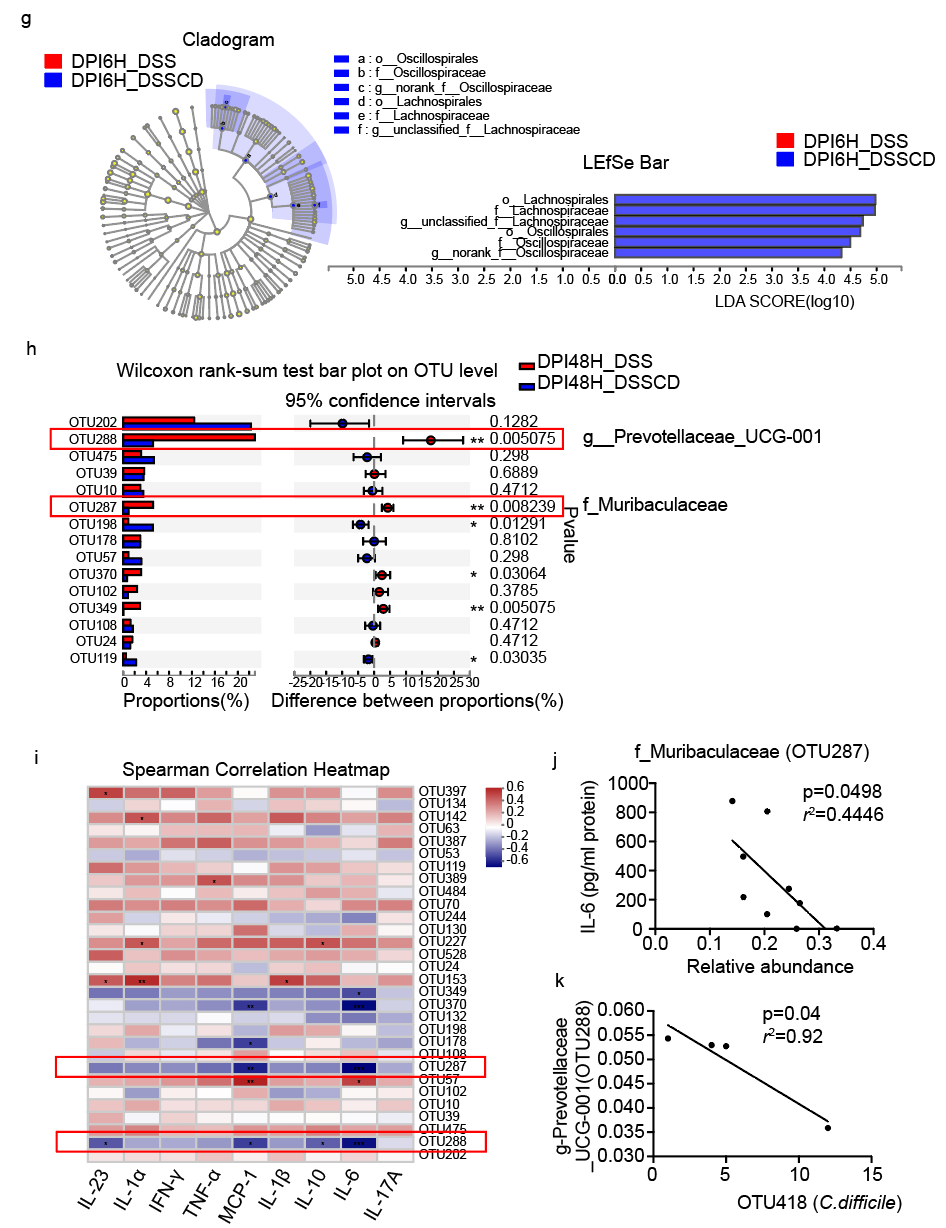


Supplementary Figure S3 (a-c) Alpha and beta diversity analysis of fecal samples between groups. Rarefaction curves of the estimated OTUs as well as the indices of Shannon and ACE index concerning bacterial (a) and fungal (c) diversity and richness respectively from each group. Each dot indicated an individual samples from each group. (b) Principle coordinated analysis (PcoA) on OTU levels in fungal communities, with plots based on the Bray-Curtis distance. The horizontal and vertical axes represented inter-sample variations, respectively. Each point represents an individual sample with different colors referring to different groups. (d) Rarefaction curves of the estimated OTUs. The horizontal and vertical axes indicated the species richness Sobs indices and OTU reads. (e,f) For colonic contents from different groups, alpha diversity analysis with Shannon and ACE indices and Faith’s phylogenetic diversity and species richness, along with PcoA analysis by weighted unifrac metrics on OTU levels. Each dot indicated an individual samples from each group. (g) Cladograms were generated by Linear discriminant analysis effect size (LEfSe) analysis to detect the differences in the bacterial taxa between DPI6_DSSCD and DPI6_DSS group. Circles presented phylogenetic levels from phylum to genus. To screen out the differentially abundant taxa, LDA threshold score was set >4.0. Red and blue colored bars indicate taxa enrichment in DPI6_DSS and DPI6_DSSCD group, respectively. (h) Wilcox rank-sum test analysis of OTUs between DPI48_DSS and DPI48_DSSCD group. The most two abundant OTUs significantly decreased in DPI48_DSSCD group were framed with red line, aligning to *g_Prevotellaceae_UCG001* and *f_Muribaculaceae* respectively. (i) heat map of correlation analysis between levels of proinflammatory cytokines and most thirty OTUs. (j and k) correlation analysis between the proportions of *f_Muribaculaceae* (OUT287) in DSS groups (DPI6H_DSS and DPI48H_DSS) and IL-6 production (j), and proportions of *g_Prevotellaceae_UCG001* and read numbers of OTU418 aligned to *C.difficile* (k). Data (a, c and e) are expressed as mean±SD. Statistical differences between groups were assessed by Mann-Whitney test. **P*<0.05, ***P*<0.01, ***P*<0.001.


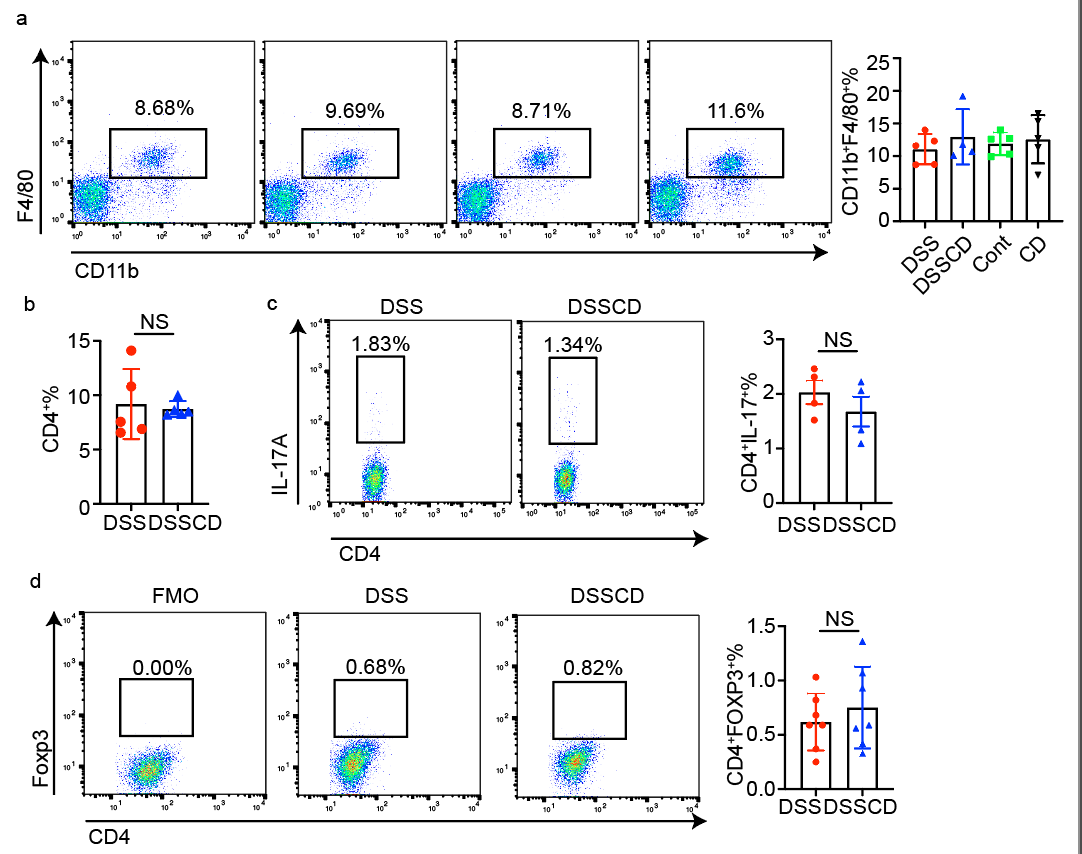


Supplementary FigureS4 (a) Representative flow plots (left) and quantification (right) of the proportions of CD11b^+^F4/80^+^ in CD45^+^ cells. (b) Proportions of CD4^+^ in CD45^+^ cells. (c, d) representative flow plots and quantification of CD4^+^IL17^+^ (c) and CD4^+^Foxp3^+^ in CD45^+^ cells. Each dot indicated an individual samples from each group. Data are expressed as mean±SD， representative of at least three independent experiments. Statistical differences between groups were assessed by Mann-Whitney test. NS, not significant.


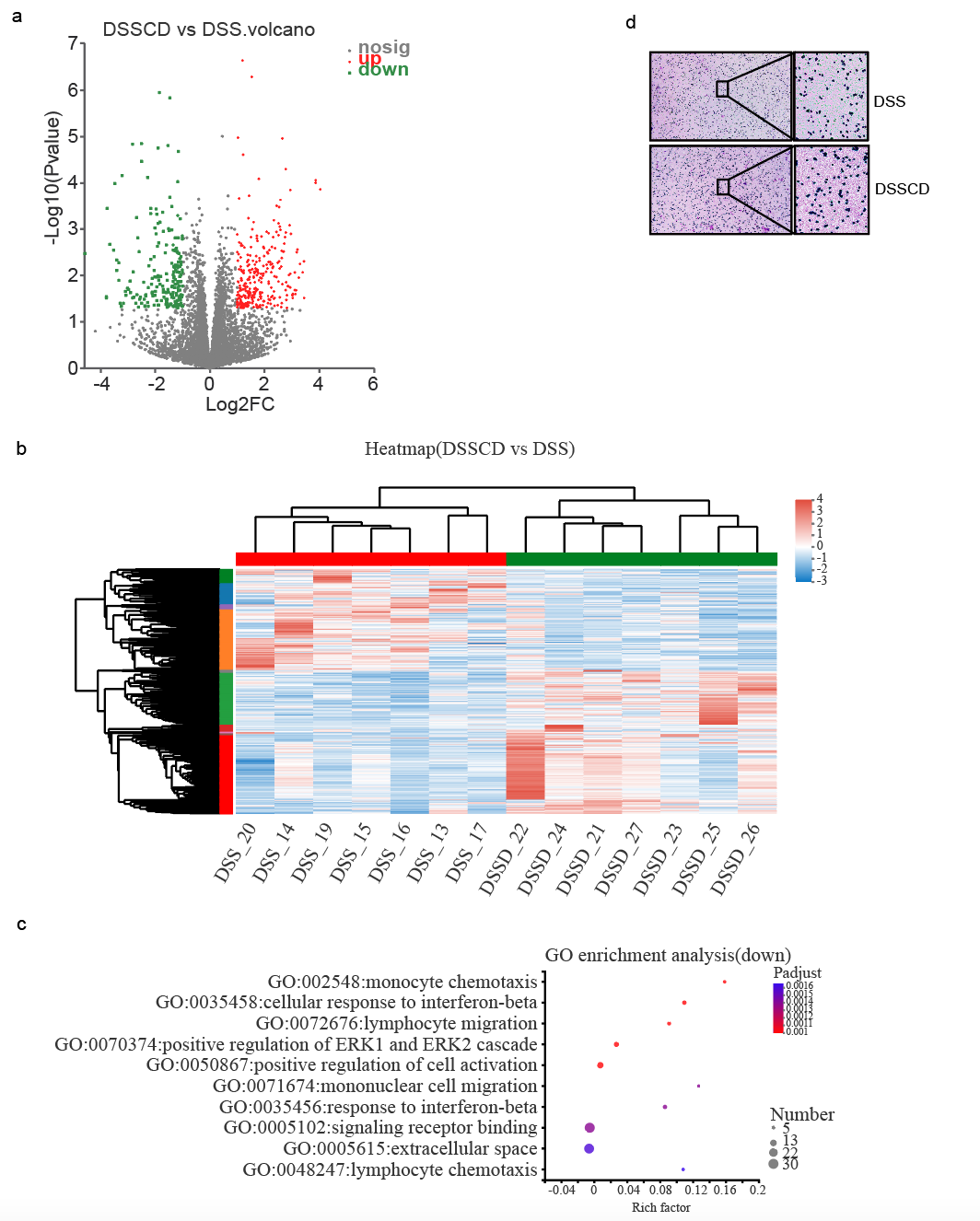


Supplementary FigureS5 (a) Volcano plot of differential expressed genes between DSSCD and DSS group. The green and red represented decreased and increased expressed in DSSCD group.

(b) heat map of differentially expressed genes in DSS (red) and DSSCD (green) group. Left panel indicated enrichment of genes. (c) down regulated genes in DSSCD mice relative DSS mice were enriched for GO functional analysis. Top 10 pathways were shown in the bubble plot. The size and color of the bubble represent gene numbers enriched in each pathway and the respective enrichment significance. (d) images of neutrophils transferred to basal membrane with violet blue staining. Magnificence, x100.


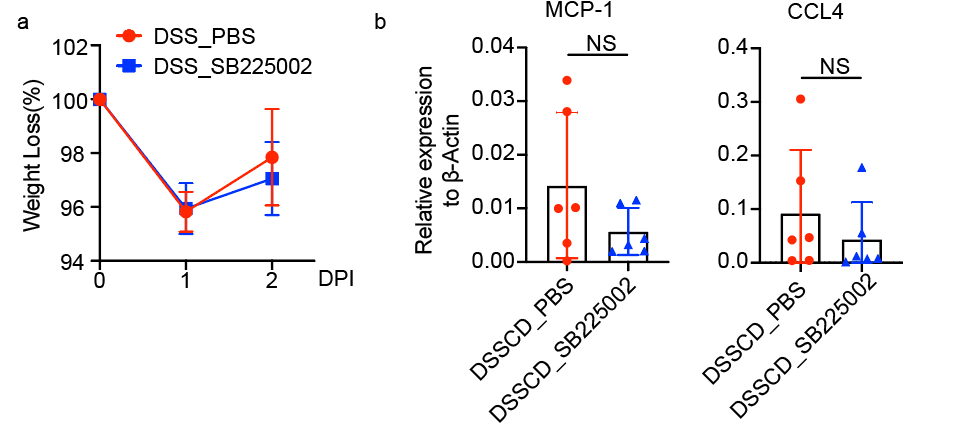


Supplementary Figure S6 (a) Weight loss of DSS mice with or without SB225002 treatment. Data are expressed as mean±SD, representative of at least three independent experiments. (b)

mRNA expressions of MCP-1 and CCL4 from colonic tissue in DSSCD mice with or without SB225002 treatment. Each dot represents an individual sample. Data are expressed as mean±SD, representative of at least three independent experiments. Statistical differences between groups were assessed by Mann-Whitney test. NS, not significant.


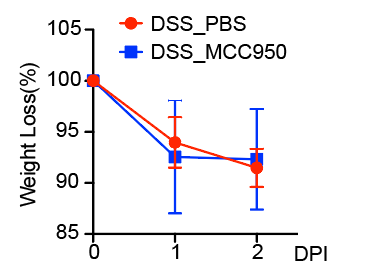


Supplementary FigureS7 Weight loss of DSS mice with or without MCC950 treatment. Data are expressed as mean±SD, representative of at least three independent experiments. Statistical differences between groups were assessed by Mann-Whitney test.
