# Supplementary material for: Clostridioides difficile aggravates dextran sulfate solution (DSS)-induced colitis by shaping the gut microbiota and promoting neutrophil recruitment
Source: Gut Microbes. 2023 Mar 23;15(1):2192478. doi: 10.1080/19490976.2023.2192478 (PMC10038061; doi:10.1080/19490976.2023.2192478)
Supplement: Supplemental Material [file KGMI_A_2192478_SM0498.zip › Supplementary table.docx]

TableS1 Primers used in the present study

| Primers | Sequences(5'-3') |
| --- | --- |
| 16S rRNA_F | CGGTGAATACGTTCCCGG |
| 16S rRNA_R | TACGGCTACCTTGTTACGACTT |
| TcdB_QPCR_F | GAAGGTGGTTCAGGTCATAC |
| TcdB_QPCR_R | CATTTTCTAAGCTTCTTAAACCTG |
| OTU288_F | TTTAAAGGGAGCGTAGGCCG |
| OTU288_R | AGCATTTCACCGCTACACCA |
| OTU287_F | TAATACGGAGGATGCGAGCG |
| OTU287_R | GCAGTTTCAACGGCTGTACG |
| mouse ꞵ-Actin_F | GGCTGTATTCCCCTCCATCG |
| mouse ꞵ-Actin_R | CCAGTTGGTAACAATGCCATGT |
| mouse Muc2_F | TGTGGAACCGGGAAGATG |
| mouse Muc2_R | GACCACAGGTATGGTTCTGGA |
| mouse Cldn2_F | CAACTGGTGGGCTACATCCTA |
| mouse Cldn2_R | CCCTTGGAAAAGCCAACCG |
| mouse Mcp-1_F | GCCTGCTGTTCACAGTTGC |
| mouse Mcp-1_R | CTTGCTGGTGAATGAGTAGCAG |
| mouse Il-6_F | TCCAATGCTCTCCTAACAGATAAG |
| mouse Il-6_R | CAAGATGAATTGGATGGTCTTG |
| mouse Il-1ꞵ_F | GATCCACACTCTCCAGCTGCA |
| mouse Il-1ꞵ_R | CAACCAACAAGTGATATTCTCCATG |
| mouse Cxcl2_F | ATGCCCTCTATTCTGCCAGAT |
| mouse Cxcl2_R | GTGCTCCGGTTGTATAAGATGAC |
| mouse Ccl4_F | TTCCTGCTGTTTCTCTTACACCT |
| mouse Ccl4_R | CTGTCTGCCTCTTTTGGTCAG |
| human ꞵ-Actin_F | CATGTACGTTGCTATCCAGGC |
| human ꞵ-Actin_R | CTCCTTAATGTCACGCACGAT |
| human Il-6_F | ACTCACCTCTTCAGAACGAATTG |
| human Il-6_R | CCATCTTTGGAAGGTTCAGGTTG |
| human Mcp-1_F | CCCCAGTCACCTGCTGTTAT |
| human Mcp-1_R | CCACAATGGTCTTGAAGATCAC |
| human Il-1ꞵ_F | ATGATGGCTTATTACAGTGGCAA |
| human Il-1ꞵ_R | GTCGGAGATTCGTAGCTGGA |
| human Cxcl2_F | CCCATGGTTAAGAAAATCATCG |
| human Cxcl2_R | CTTCAGGAACAGCCACCAAT |
| human Ccl4_F | GCTGCCTTCTGCTCTCTAGC |
| human Ccl4_R | ACCACAAAGTTGCGAGGAAG |
